# Cancer‐associated fibroblasts educate normal fibroblasts to facilitate cancer cell spreading and T‐cell suppression

**DOI:** 10.1002/1878-0261.13077

**Published:** 2021-11-05

**Authors:** Go Itoh, Kurara Takagane, Yuma Fukushi, Sei Kuriyama, Michinobu Umakoshi, Akiteru Goto, Kazuyoshi Yanagihara, Masakazu Yashiro, Masamitsu Tanaka

**Affiliations:** ^1^ Department of Molecular Medicine and Biochemistry Akita University Graduate School of Medicine Japan; ^2^ Department of Life Science Faculty and Graduate School of Engineering and Resource Science Akita University Japan; ^3^ Department of Cellular and Organ Pathology Akita University Graduate School of Medicine Japan; ^4^ Division of Biomarker Discovery Exploratory Oncology Research & Clinical Trial Center National Cancer Center Kashiwa Japan; ^5^ Department of Surgical Oncology Osaka City University Graduate School of Medicine Japan

**Keywords:** asporin, CAF‐educated fibroblasts, CD8^+^ T cells, IGF‐I, kynurenine

## Abstract

In some tumors, a small number of cancer cells are scattered in a large fibrotic stroma. Here, we demonstrate a novel mechanism for expansion of pro‐tumor fibroblasts via cancer‐associated fibroblast (CAF)‐mediated education of normal fibroblasts (NFs). When NFs were incubated with conditioned medium from CAFs, the resulting CAF‐educated fibroblasts (CEFs) generated reactive oxygen species, which induced NF‐κB‐mediated expression of inflammatory cytokines and the extracellular matrix protein asporin (ASPN), while expression of a common CAF marker gene, α‐SMA, was not increased. ASPN further increased CEF expression of downstream molecules, including indoleamine 2,3‐dioxygenase 1 (IDO‐1), kynureninase (KYNU), and pregnancy‐associated plasma protein‐A (PAPP‐A). These CEFs induce cytocidal effects against CD8^+^ T cells and IGF‐I activation in cancer cells. CEFs were generated without cancer cells by the direct mixture of NFs and CAFs in mouse xenografts, and once CEFs were generated, they sequentially educated NFs, leading to continuous generation of CEFs. In diffuse‐type gastric cancers, ASPN^high^/IDO‐1^high^/KYNU^high^/α‐SMA^−^ CEFs were located at the distal invading front. These CEFs expanded in the fibrotic stroma and caused dissemination of cancer cells. ASPN may therefore be a key molecule in facilitating tumor spreading and T‐cell suppression.

## Graphical abstract

Cancer‐associated fibroblast (CAF)‐educated fibroblasts (CEFs), a newly identified source of pro‐tumor fibroblasts, were generated from normal fibroblast (NFs) by CAF‐mediated education. CAFs activated reactive oxygen species production in NFs, reprogramming them to secrete inflammatory cytokines and ASPN. ASPN further induced expression of IDO‐1, KYNU, and PAPP‐A in CEFs, leading to cancer cell dissemination, CD8^+^ T‐cell cytotoxicity, and activation of IGF‐I signaling in cancer cells.

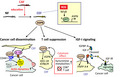

**Abbreviations**
ASPN: asporin
CAFs: cancer‐associated fibroblasts
CEFs: CAF‐educated fibroblasts
CM: conditioned media
EMT: epithelial–mesenchymal transition
FAP: fibroblast activation protein
FGF: fibroblast growth factor
HIF: hypoxia‐inducible factor
IDO‐1: indoleamine 2, 3‐dioxygenase 1
IGF: insulin growth factor
KYN: kynurenine
KYNU: kynureninase
NFs: normal fibroblasts
PAPP‐A: pregnancy‐associated plasma protein‐A
ROS: reactive oxygen species
siRNA: small‐interfering RNA
SMA: α‐smooth muscle activation
SPF: specific pathogen‐free
TGF: transforming growth factor
TME: tumor microenvironment
α‐SMA: alpha‐smooth muscle actin

## 1 Introduction

Cancer‐associated fibroblasts (CAFs) play important roles in tumor progression by inducing cancer cell proliferation, invasion, and metastasis [1, 2]. While the origins of CAFs remain controversial, it has been proposed that resident normal fibroblasts (NF) differentiate into CAFs through education by cancer cells [3]. CAFs are also reported to be derived from pluripotent cells including mesenchymal stem cells of the bone marrow [4, 5]. Although this education mechanism is incompletely understood, cancer cell‐released cytokines, including transforming growth factor (TGF)‐β and fibroblast growth factor (FGF)2, and factors contained within extracellular vesicles activate quiescent fibroblasts and induce expression of CAF marker genes, including α‐smooth muscle activation (SMA) and fibroblast activation protein (FAP) [6, 7, 8].

Because fibrosis is sometimes observed in large tumor areas beyond the regions in which cancer cell nests reside, we hypothesized that pro‐tumor fibroblasts could be generated in the absence of direct education by cancer cells. In the present study, we identified a novel mechanism of fibroblast activation, in which CAFs educated NFs to generate pro‐tumor fibroblasts, which we refer to as CAF‐educated fibroblasts (CEFs). Moreover, once CEFs were generated, they successively educated NFs to generate new CEFs.

Recent studies have revealed that spatially and functionally distinct subclasses of fibroblasts are present in the tumor microenvironment (TME). For example, single‐cell RNA sequencing of mesenchymal cells from a mouse tumor model and human tumor specimens identified distinct subpopulations of CAFs that harbor distinct transcriptional profiles [9, 10]. In addition to pro‐tumor CAFs, some types of CAFs prevent tumor progression [11, 12]. CAF heterogeneity refers to the presence of at least two major subtypes of pro‐tumor CAFs: inflammatory CAFs (iCAFs), which express high levels of inflammatory cytokines and chemokines (such as CXC motif chemokine ligand (CXCL)1 and CXCL8 [13]), and myofibroblastic CAFs (myoCAFs), which express high level of α‐SMA in response to TGF‐β signaling [14]. In addition, an immunosuppressive subset of CAFs that increases the generation of regulatory T cells has been reported [15].

We previously identified asporin (ASPN), a small leucine‐rich proteoglycan, as the major extracellular matrix protein secreted by CAFs in gastric cancer [16]. In various tumors, ASPN is expressed primarily by stromal fibroblasts, which are positively associated with malignancy in pancreatic, colorectal, gastric, and prostate cancers [17, 18, 19]. ASPN also plays dual roles in breast cancer with both pro‐ and anti‐tumor effects reported [20]. In gastric cancer, ASPN‐positive CAF‐like cells did not largely overlap with α‐SMA^+^ CAFs, suggesting that ASPN^+^ fibroblasts have distinct functions in the TME. The present study revealed that ASPN may play a central role in CEFs, which promote cancer cell dissemination, T‐cell suppression, and insulin growth factor (IGF)‐I signaling.

We observed that CAF‐conditioned media (CM), as well as direct mixture of NFs with CAFs, induce CEF generation from NFs via reactive oxygen species (ROS)‐induced activation of nuclear factor NF‐κB signaling, resulting in the induction of a pro‐inflammatory loop and ASPN secretion. Moreover, ASPN further activated IGF‐I signaling and the tryptophan metabolic pathway, leading to T‐cell death. Inflammatory cytokines, ASPN, and downstream molecules were successively transmitted from fibroblast to fibroblast via the generation of CEFs, ultimately resulting in cancer cell spreading and tumor dissemination. Our observations suggest that CEFs are a newly identified mediator in the rapid expansion of pro‐tumor fibroblasts. Furthermore, our findings suggest that blocking sustained CEF generation may be a potential therapeutic strategy for the prevention of immunosuppression and tumor dissemination.

## 2 Materials and methods

### 2.1 Cells

OCUM‐12, 44As3, and HSC‐43 cells were isolated from patients with scirrhous gastric carcinoma [21, 22]. OCUM‐12 was donated by M. Yashiro (Osaka City University Medical School), and 44As3 and HSC‐43 were from K. Yanagihara (National Cancer Center). Li‐7, hepatocellular carcinoma line, was obtained from RIKEN BRC. All cells were screened for mycoplasma and maintained in culture for less than 6 months after receipt. Cancer cells were cultured in RPMI‐1640 medium supplemented with 10% FBS. Human CAFs were obtained from the tumoral gastric wall, and NFs were obtained from the noncancerous gastric wall of the same patient. The tissues were excised under aseptic conditions and minced with forceps and scissors. The pieces were cultured in DMEM supplemented with 10% FBS, 100 IU·mL^−1^ penicillin, 100 μg·mL^−1^ streptomycin, and 0.5 mm sodium pyruvate, and incubated at 37 °C in 5% CO_2_. After approximately 2 weeks, fibroblasts were collected and transferred to other culture dishes. Cells were then serially passaged every 4–7 days [23]. CAFs and NFs derived from three scirrhous gastric cancer patients (patient number: #50, #51, and #54) were used, and each CEF was generated by the NF and CAF from the same patient. NFs and CAFs were obtained from Osaka City University Medical School. CAFs, NFs, and CEFs were cultured in DMEM containing 4500 mg·mL^−1^ glucose, 1 mm sodium pyruvate, and 10% FBS. The study complied with the Declaration of Helsinki and was approved by the Osaka City University Ethics Committee (approval number 2756, Osaka, Japan) and Akita University Ethics Committee (approval number b‐1‐3159, Akita, Japan). All patients provided informed consent prior to the study.

### 2.2 Antibodies and reagents

The following antibodies were purchased from Santa Cruz Biotechnology (Dallas, TX, USA): anti‐IDO (sc‐376413), anti‐IGF‐I (sc‐74116), anti‐IGFBP‐4 (sc‐517440), anti‐IGF‐IR (sc‐81464), anti‐Kynurenine (sc‐69890), anti‐Smad2/3 (sc‐6032), anti‐PAPP‐A (for immunoblotting, sc‐365226), from Cell Signaling Technology (Danvers, MA, USA): anti‐phospho‐AKT (9271S), E‐cadherin (3195), anti‐COX2 (12282), anti‐phospho‐ERK (9101S), anti‐ERK (9102), anti‐Phospho‐IGF‐1Rβ (3024), anti‐phospho‐IKBα (2859), anti‐phospho‐IKKa/B (2697), NF‐κB p65 Antibody Sampler Kit (4767), anti‐vimentin (3932S), anti‐phospho‐Smad2 (3108S), anti‐Snail (3879), anti‐phospho‐p38 (9211S), anti‐p38 (9212), from Sigma‐Aldrich (St. Louis, MO, USA): anti‐KYNU (HPA031686), anti‐LIF (HPA018844), anti‐α‐tubulin (T5168), from Abcam (Cambridge, UK): anti‐CXCL5+CXCL6 (EP13083) (ab198505), anti‐IL‐1B (ab9722), or from Proteintech (16806‐1‐AP, Chicago, IL, USA): anti‐CST1 (16025‐1‐AP), anti‐IL‐33 (66235‐1), anti‐KMO (10698‐1‐AP), and anti‐PAPP‐A (66962‐1‐Ig, for immunostaining). Other antibodies were anti‐Ki67 (MIB‐1; DAKO, Santa Clara, CA, USA), anti‐α‐SMA (IA4; ScyTek, Cache Valley, UT, USA), anti‐Rac1 (BD Bioscience, Franklin Lakes, NJ, USA, 610651), anti‐N‐cadherin (BD Transduction, Franklin Lakes, NJ, USA, 610920), anti‐HIF‐1 alpha (GTX127309; GeneTex, Los Angeles, CA, USA), anti‐HA Tag (MMS‐101R; Berkeley Antibody, Berkeley, CA, USA), anti‐ASPN (for immunoblot; Sigma‐Aldrich HPA008435, for immunohistochemistry; Abcam ab58741). EZ‐Link Sulfo‐NHS‐LC‐biotin (membrane‐impermeable) was obtained from Thermo Fisher Scientific (Waltham, MA, USA). Recombinant proteins were human IL‐1B (PeproTech, Rocky Hill, NJ, USA), asporin (H00054829‐P01; Novus, Centennial, CO, USA), and TGF‐β (R & D Systems, Minneapolis, MN, USA). Reagents were *N*‐acetylcysteine (NAC) (Abcam), NLG‐919 (Abcam), NSC23766 (Abcam), Y‐27632 (Sigma‐Aldrich), Echinomycin (AdipoGen Life Sciences, Basel, Switzerland), Withaferin A (AdipoGen Life Sciences), NS‐398 (Selleck Chemicals, Houston, TX, USA), and GM6001 (Cayman Chemical, Ann Arbor, MI, USA).

### 2.3 Gene expression profiling by mRNA microarray analysis

RNAs purified from NFs, CEFs, and CAFs were subjected to Clariom S microarray analysis (Filgen, Nagoya, Japan). Data were analyzed using the Microarray Data Analysis Tool Ver3.2 (Filgen) and DAVID Bioinformatics Resources 6.8 (Laboratory of Human Retrovirology and Immunoinformatics, Frederick, MD, USA).

### 2.4 Quantitative real‐time PCR analysis and RT‐PCR

Total RNA was extracted from the indicated cells using an RNeasy Mini Kit (Qiagen, Dusseldorf, Germany), and cDNA was produced from 1 µg RNA by reverse transcription, according to the manufacturer's recommendations (Roche, Mannheim, Germany). Quantitative PCR was conducted in a LightCycler Nano using a SYBR Green kit (Roche Diagnostics, GmbH, Basel, Switzerland). Samples were normalized against GAPDH. A list of primers is summarized in Table S1.

### 2.5 Transfection, viral infection, and siRNA

Human cDNA encoding H2BJ and synthetic DNA fragment of MyrPalm (myristoylated and palmitoylated) were cloned into pEGFP‐N3 (Clontech, Santa Clara, CA, USA). Human cDNA encoding asporin (variant 1, corresponding to GenBank NM_017680) was cloned into the pCSII‐CMV‐MCS vector (RIKEN, Saitama, Japan). Expression vector was transfected using Lipofectamine 2000 according to the manufacturer's instructions (Life Technologies, Waltham, MA, USA). OCUM‐12 cells stably expressing EGFP, MyrPalm‐EGFP, or H2BJ‐EGFP vector were established by selection in G418‐containing medium after transfection. IGFBP‐4‐tagged HA was transiently expressed in COS‐1 cells. Recombinant lentiviral plasmids were co‐transfected along with packaging vectors into 293T cells to allow the production of the viral particles. NF cells and HSC‐43 cells stably expressing ASPN cDNA were established after viral infection by selection in medium containing puromycin. The selected cells were collected and used in bulk. To knockdown of ASPN in HSC‐43 ASPN cells, small‐interfering RNA (siRNA) of ASPN was synthesized as follows: Sense‐1, 5′‐CAGUGCCAAAGAUGAAGAAAUCUUU‐3′; Sense‐2, 5′‐UGAAGGAGUAUGUGCUCCUAUUAUU‐3′. Scramble II duplex (Dharmacon, Lafayette, CO, USA) was used as a nontargeting control siRNA that does not possess homology with known gene targets in mammalian cells. siRNAs were incorporated into cells with Lipofectamine RNAiMAX (Invitrogen, Carlsbad, CA, USA). Assays were performed at 72 h post‐treatment.

### 2.6 Immunoblotting

Cell lysates were prepared in PLC buffer [50 mm HEPES (pH 7.5), 150 mm NaCl, 1.5 mm MgCl_2_, 1 mm EGTA, 10% glycerol, 100 mm NaF, 1 mm Na_3_VO_4,_ and 1% Triton X‐100] containing protease inhibitors. Cell lysates were separated by SDS/PAGE and immunoblotted. All primary antibodies were used at dilutions of 1 : 1000. Band intensities were quantified using the fiji software, and the abundance of protein was normalized against α‐tubulin.

### 2.7 Rac1‐GTP pull‐down assay

The activation of Rac1 was monitored by affinity precipitation of GTP‐bound Rac1 with a GST‐fusion of the p21‐binding domain of PAK1 (GST‐PBD). Briefly, cell lysates were prepared in lysis buffer [50 mm HEPES (pH 7.5), 150 mm NaCl, 10 mm MgCl_2_, 10% glycerol, 100 mm NaF, 1 mm Na_3_VO_4,_ and 1% Triton X‐100] and then incubated for 45 min at 4 °C with glutathione‐Sepharose beads containing GST‐PBD. Precipitates were washed four times in the same buffer, and the precipitated GTP‐bound Rac1 was detected by immunoblotting.

### 2.8 PAPP‐A proteolytic assay

The proteolytic activity of PAPP‐A derived from fibroblasts was evaluated by cleavage of the substrate protein IGFBP‐4. Briefly, for replicate reactions, a mixture of COS‐1 cell lysate containing IGFBP‐4 tagged with HA at the C terminus and recombinant human IGF‐I (75 nm final concentration) were incubated at 4 °C for 2 h to allow binding of IGF‐I and IGFBP‐4, and the mixture was subsequently added to fibroblast cell lysate and incubated at 37 °C for 2 h to allow for IGFBP‐4 cleavage. IGFBP‐4‐HA cleavage was detected by immunoblotting using anti‐HA antibody. Activation of IGF‐IR in cancer cells by liberation of IGF‐I was examined as follows. Recombinant human IGF‐I was incubated with COS‐1 medium containing IGFBP‐4 at 4 °C for 2 h and subsequently added to CEFs and further incubated at 37 °C for 2 h. The medium was collected and transferred to a dish containing cultured cancer cells. Activation of IGF‐IR was evaluated by immunoblotting of cancer cell lysate with anti‐phospho‐IGF‐IRβ antibody.

### 2.9 Specimens from cancer patients

Gastric cancer specimens were obtained from patients who had undergone resection of primary gastric tumors. None of the patients had undergone preoperative radiation or chemotherapy. All samples were collected from the surgical pathology files at Akita University Hospital, Akita, Japan, between 2008 and 2013, and tissues were obtained with the informed consent of the patients. The study was approved by the Akita University Ethics Committee (approval number 1662, Akita, Japan).

The experiments were undertaken with the understanding and written consent of each subject. The study methodologies conformed to the standards set by the Declaration of Helsinki.

### 2.10 Mice

Specific pathogen‐free (SPF) BALB/c^nu/nu^ nude mice and SPF C57BL/6JJcl mice were obtained from CLEA Japan, Inc. (Tokyo, Japan). Mice were bred under SPF conditions at the Animal Research Laboratory Bioscience Education Research Center of Akita University. Six‐week‐old female mice were used for inoculation of cancer cells. All animal protocols were approved by the Committee for Ethics of Animal Experimentation (approval number a‐1‐3175), and all experiments were conducted in accordance with the Guidelines for Animal Experiments.

### 2.11 *In vivo* tumor transplantation

All animal experimental protocols were approved by the Committee for Ethics of Animal Experimentation, and the experiments were conducted in accordance with the guidelines for Animal Experiments at Akita University. Biotin‐labeled NF alone (1 × 10^6^ cells) or a mixture of CAF and labeled NF (5 × 10^5^ of each cell type) was injected into the subcutaneous tissue of six‐week‐old female BALB/c nude mice (CLEA Japan, Inc, Shizuoka, Japan). The mice were euthanized 2 days after injection. Three mice were used. DiI‐labeled NF (3 × 10^5^), CAF (2 × 10^5^), or the mixture of labeled NF and CAF were injected into the pancreases of nude mice together with OCUM‐12 cells expressing EGFP (1 × 10^5^). 5 mg·kg^−1^ COX2 inhibitor NS‐398 was injected intraperitoneally daily. The mice were euthanized 14 days after injection. Five mice were used per group. DiI‐labeled NF (2 × 10^5^) or CEF (2 × 10^5^) were injected into the gastric wall of nude mice together with OCUM‐12 cells (2 × 10^5^). The mice were euthanized 8 days after injection. Five mice were used per group.

### 2.12 Immunohistochemical analysis

Paraffin blocks were sectioned and subjected to immunohistochemical staining using the Envision reagent (Dako). Antigen retrieval was performed by placing sections in Target retrieval solution (Dako) and heating to 95 °C in a water bath, according to the manufacturer's instructions. In co‐immunostaining experiments, sections were sequentially stained with each antibody using an Opal™ four‐color IHC Kit and fluorescently conjugated tyramide according to the manufacturer's instructions (PerkinElmer, Waltham, MA, USA). All primary antibodies were used at dilutions of 1 : 500. Horseradish peroxidase‐conjugated secondary antibody (GE Healthcare, Chicago, IL, USA) was added for 10 min and incubated with Opal kit working solution including the desired fluorophore. Tissues underwent the microwave treatment for removal of primary and secondary antibodies before another round of staining according to the Opal Multiplex IHC Assay Development Guide and Image Acquisition Information (Akoya Biosciences, Tokyo, Japan).

### 2.13 Immunofluorescence staining

Cells were fixed with 4% paraformaldehyde in PBS and permeabilized for 5 min with 0.1% Triton X‐100. Cells were preincubated in 3% bovine serum albumin for 30 min and incubated with specific primary antibodies (1 : 500 dilution) for 3 h at room temperature. After washing, cells were incubated with Alexa Fluor‐conjugated secondary antibodies (Invitrogen) for 1 h at room temperature. Images were obtained using an LSM780 (Zeiss, Oberkochen, Germany) confocal microscope and processed using zen software (Zeiss).

### 2.14 Evaluation of mtROS

To evaluate mtROS, MitoSOX^®^ Red (Thermo Scientific, Waltham, MA, USA), a mitochondrial superoxide indicator, was added to living cells at 5 μm according to the manufacturer's instructions. Labeled cells were fixed, stained with 4′, 6′‐diamidino‐2‐phenylindole (DAPI), and the images were obtained using an LSM780 (Zeiss) confocal microscope. To detect mtROS in mice xenografts, excised tissues were 200‐μm sliced using a LinearSlicer (Dosaka EM, Kyoto, Japan) and immediately labeled by MitoSOX^®^ Red in culture medium. The tissues were fixed and frozen‐sectioned for imaging.

### 2.15 Flow cytometric analysis and cell sorting

CD8^+^ T cells were purified from C57BL/6JJcl mouse spleens using MojoSort isolation kits (480008 BioLegend, San Diego, CA, USA). CD8^+^ T cells (2 × 10^6^) were incubated with CM derived from fibroblasts or cancer cells for 2 days. CD8^+^ T cells were overlayed on a sheet of fibroblasts and cocultured for 2 days. After incubation, CD8^+^ T cells were labeled with the following mouse antibodies: phycoerythrin (PE)‐conjugated CD8a (Miltenyi Biotec, Bergisch Gladbach, Germany), FITC‐conjugated Annexin‐V (Miltenyi Biotec). Propidium iodide (PI) (BD Biosciences, San Jose, CA, USA) and 7‐aminoactinomycin D (7‐AAD) (Miltenyi Biotec) were used to identify dead cells. Cells were subjected to FACS analysis using a BD FACSAriaTM III (BD Biosciences) with FACSDiva and bd flowjo software (BD Biosciences). To isolate biotin‐labeled fibroblasts, cells were stained with FITC‐conjugated streptavidin and sorted (FACSAria III; BD Biosciences, San Jose, CA, USA). Cell contamination was eliminated using FSC‐H and FSC‐W, SSC‐H, and SSC‐W. For positive and negative populations, the top 25% of stained cells or the bottom 20% of unstained cells were selected to be sorted, respectively. After collecting, the purity of the cell fraction was confirmed by FITC fluorescence or immunoblotting.

### 2.16 ELISA

IL‐1β protein level in fibroblast‐conditioned medium (CM) was measured using Human IL‐1β ELISA kit according to the manufacturer's instructions (Proteintech). Anti‐IL‐1β antibody provided by the kit recognizes both mature and immature IL‐1β protein. CM derived from 5 × 10^5^ cells was collected after incubation in FBS‐free fresh medium for 24 h. The concentration of IL‐1B in each sample was calculated from the standard curves. Kynurenine secreted in the CM of fibroblasts or HSC‐43 cells was quantified by l‐Kynurenine ELISA kit according to the manufacturer's instructions (ImmuSmol, Bordeaux, France). CM derived from 2 × 10^5^ cells of each was collected after incubation in medium containing 0.5% FBS and 50 μm l‐Tryptophan for 72 h.

### 2.17 Separation of biotin‐NF by microbeads

Positive selection of biotin^+^ NFs was performed using microbeads. Cells were magnetically labeled with streptavidin microbeads (Miltenyi Biotec) and passed through MACS MS columns (Miltenyi Biotec), which were placed in the magnetic field according to the manufacturer's instruction. Biotin^+^ NFs were flushed out from the column and passed over a new column to increase the purity.

### 2.18 Time‐lapse imaging

Dynamics of OCUM‐12 cells expressing H2BJ‐EGFP were analyzed by time‐lapse imaging, which began directly after replacing growth medium with fibroblast‐CM (1 : 1 mixture of CM and fresh complete medium). Fluorescent images were captured by time‐lapse microscopy with the 10× objective at 10 min intervals for 20 h (37 °C, 5% CO_2_; BZ‐9000, Keyence, Osaka, Japan). Distribution of OCUM‐12 cells was analyzed using fiji software as described previously [24]. Briefly, the value of *x*–*y* axes from the nuclear position was tracked using the Manual Tracking plugin in fiji software. The average velocity of cell migration was calculated by dividing the trajectory distance from the tracked data and the capture time.

### 2.19 Delaunay triangulation

The center of the nuclei was automatically detected using the Particle analysis function of imagej software (NIH, Bethesda, MD, USA). Then, the *xy* positions were used to generate Delaunay triangulation plots. The Delaunay plots were computed using the python3, numpy, scipy, and matplot functions on the Anaconda3 platform. The area of each triangle was extracted, and the areas of the regular triangles in each plot were used for normalization. The cobblestone‐like structures of cellular sheets are described as regular triangles because the distances between nuclei are usually uniform. When cell dispersion disturbs the position of the cells, then triangles take on an irregular form. Therefore, the expansion of triangular areas describes the local dispersion of a cellular sheet. The average area of the Delaunay triangles was plotted and analyzed by one‐way ANOVA [25].

### 2.20 Boyden chamber assays

Migration assays were performed using Boyden chambers with a polycarbonate nucleopore membrane (Falcon, 8 µm pore size). First, 2 × 10^4^ cells in 200 µL serum‐free medium were seeded onto the upper part of each chamber, and the lower comportment was filled with a mixture of 400 µL fresh medium and 400 µL fibroblasts‐derived conditioned medium containing 1.0% FBS. After incubation for 20 h at 37 °C, nonmigrated cell on the upper surface of the filter was removed by a cotton swab, and the migrated cells on the lower surface of the filter were fixed and stained with Giemsa stain solution. The total number of migrated cells was counted in four microscopic fields per well, and average value was calculated.

### 2.21 Statistical analysis

All the data in our study were based on three independent experiments at least and exhibited mean ± standard deviations. Statistical significance was calculated using Student's *t*‐tests (*P < 0.05, **P < 0.01, ***P < 0.001) or one‐way ANOVA followed by Tukey's *post hoc* tests (*P < 0.05, **P < 0.01, ***P < 0.001). *P* < 0.05 was considered statistically significant.

## 3 Results

### 3.1 CAF‐conditioned medium activated NFs

**Fig. 1:**
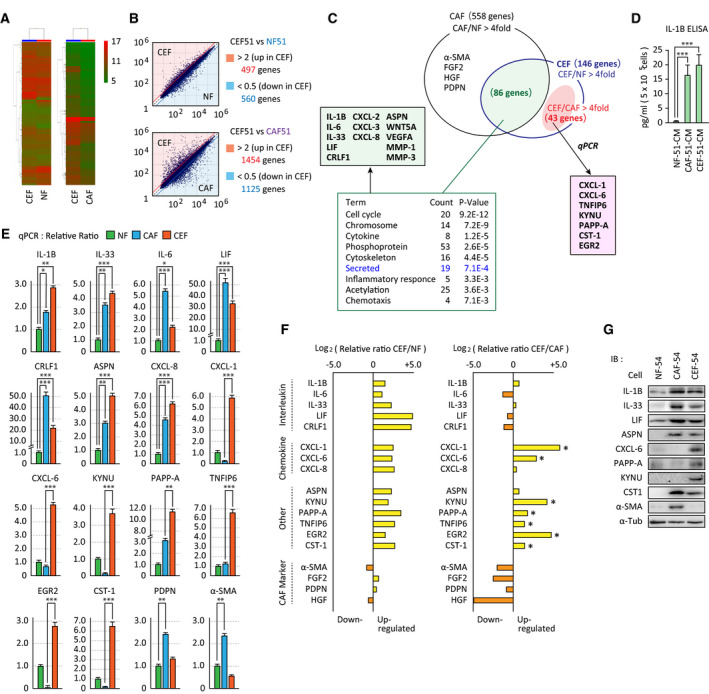
Gene expression profiling of CEFs. (A–C) RNA was purified from NF‐51, CAF‐51, and CEF‐51 cells, and subjected to microarray analysis. (A) Heat maps showing the comparison between CEFs and NFs, or CEFs and CAFs. (B) Scatter plot of the genes in CEF/NF and CEF/CAF. (C) Venn diagram of differentially expressed genes in CAF‐51 and CEF‐51 cells. Upregulated genes in CAFs or CEFs compared with NFs (more than 4.0‐fold) were classified. GO analysis with 86 shared genes in CAFs and CEFs (intersection) was shown at the bottom, in which representative genes of ‘Secreted’ were listed (green box). Genes exclusively upregulated in CEFs (CEF > CAF, NF) were validated by qRT‐PCR and listed in the red box. (D) The amount of IL‐1β in CM from NF‐51, CAF‐51, and CEF‐51 cells was examined by ELISA. ELISA was performed using CM samples derived from equal numbers of cells (5 × 10^5^). ***P < 0.001. The data in bar charts are presented as the mean ± SD. (E) Validations of gene tip analyses by qRT‐PCR. Results are expressed as the relative ratio to NFs. (F) Summary of gene expressions validated by qRT‐PCR; upregulated (yellow columns) or downregulated (orange) in CEFs compared with NFs (left) or CAFs (right). Asterisk indicated seven genes exclusively increased in CEFs. (G) Immunoblot analysis of representative genes in E. (E–G) Assays were performed using NF/CAF/CEF prepared from patients #50, #51, and #54, and representative results consistently observed in both lines are shown. Statistical significance was calculated using a one‐way ANOVA followed by Tukey's *post hoc* tests (*n* = 3, *P < 0.05, **P < 0.01, ***P < 0.001). The data in bar charts are presented as the mean ± SD.

To examine the possibility that heterogeneous cancer‐promoting fibroblasts are generated in the TME via CAF education of NFs, gastric NFs were incubated with CM of CAFs obtained from gastric cancer patients. RNA isolated from NFs after treatment with CAF‐CM was compared with RNA from untreated NFs and CAFs by microarray analysis (Fig. 1A). We detected 497 upregulated genes (> 2‐fold) and 560 downregulated genes (< 0.5‐fold) between CAF‐CM‐educated NFs (CEFs) and untreated NFs (Fig. 1B, top). In addition, 1454 upregulated genes (> 2‐fold) and 1125 downregulated genes (< 0.5‐fold) were identified between CEFs and CAFs (Fig. 1B, bottom). Upregulated genes in CAFs or CEFs compared with NFs (> 4.0‐fold) were classified into three groups (Fig. 1C). Gene ontology (GO) analysis was performed on the 86 commonly upregulated genes in CAFs and CEFs relative to NFs, which identified 19 genes classified as ‘secreted’ (Fig. 1C intersection). In particular, expression of the IL‐1 superfamily members *Il‐1b* and *Il‐33*; the IL‐6 superfamily members *IL‐6*, *Lif,* and *Crlf1*; the cytokines *Wnt5A* and *VegfA*; and the chemokines *Cxcl2*, *Cxcl3,* and *Cxcl8* was commonly upregulated in CAFs and CEFs. Additionally, expression of *ASPN,* which encodes an extracellular substrate in gastric cancer, and other inflammatory response genes (*Mmp‐1*, *Mmp‐3*) was increased in both CEFs and CAFs (Fig. 1C, green box). Conversely, transcript levels of genes encoding cytokines known to be highly expressed in CAFs (*Hgf*, *Fgf2*) and the common CAF markers α‐SMA and podoplanin (*Pdpn*) were far less elevated in CEFs than in CAFs (Fig. 1C). These findings suggested that CEFs have an inflammatory phenotype with some characteristics of CAFs. Increased secretion of IL‐1β protein in CEF‐CM was further confirmed by ELISA (Fig. 1D).

Next, expression of the genes described above and of 43 selected genes that were specifically increased in CEFs compared with untreated NFs and CAFs was validated by quantitative real‐time PCR (qRT‐PCR) using mRNA isolated from NF, CAF, and CEF prepared from two different patients. Expression of seven genes increased in CEFs relative to NFs and CAFs in both patients, including *CXCL‐1*, *CXCL6, KYNU, PAPP‐A, TNFIP6, EGR2*, and *CST1* (Fig. 1C red box, E). A summary of the validation genes at the protein level is shown in Fig. 1F. Among these, CXCL6, KYNU, and PAPP‐A, latter two are involved in tryptophan metabolism and IGF‐I signaling and were the most highly elevated at the protein level in CEFs (Fig. 1G).

Taken together, these results indicated that CEFs are transcriptionally distinct from myoCAF and acquired unique expression of CXCL6, KYNU, and PAPP‐A, as well as expression of inflammatory modulators and ASPN.

### 3.2 CEFs were generated by mixture of NFs with CAFs *in vitro* and *in vivo*

We subsequently determined if direct mixture of NFs with CAFs generated CEFs *in vitro*, under standard culture conditions, or *in vivo*, in mouse xenografts. NFs were labeled with membrane‐impermeable biotin and mixed with CAFs, and induction of CEF marker genes was assessed in biotin‐labeled NFs.

**Fig. 2:**
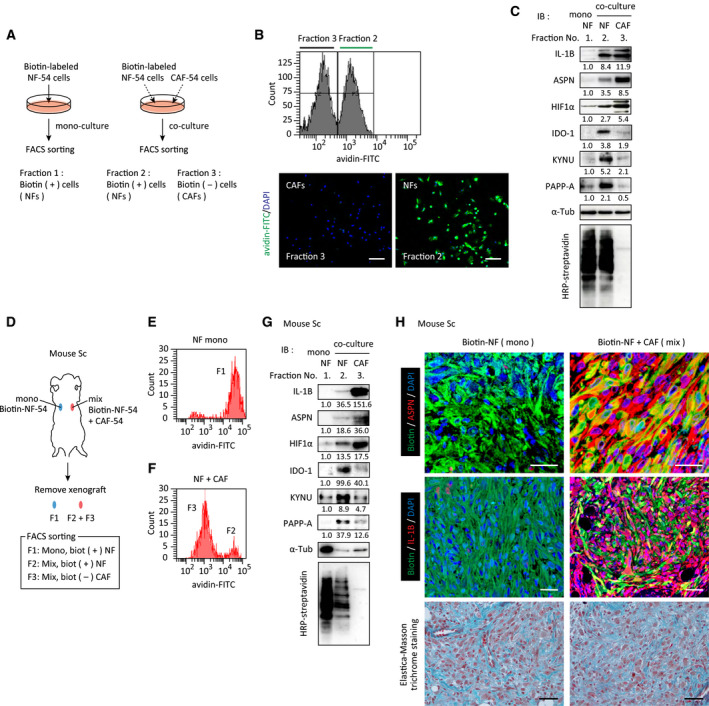
CEFs were generated from NFs by their mixture with CAFs *in vitro* and *in vivo*. (A) Schematic of the experiment. Cell surface proteins of NF‐54 cells were labeled with membrane‐impermeable biotin. Biotin‐NF‐54 cells and unlabeled CAF‐54 cells were mixed at 1 : 1 ratio and cocultured for 2 days. Cells were collected and stained by FITC‐streptavidin and sorted as biotin^+^ cells (fraction 2; NF) and biotin^−^ cells (fraction 3; CAF) using a flow cytometer (B, top). The experiments were repeated for three times, and representative images of each fraction are shown (B, bottom). Scale bar = 100 μm. As a control, biotin‐NFs were monocultured (fraction 1). Cells from each fraction were lysed and subjected to immunoblot analysis (C). The number below the panel indicates the ratio of the band intensity to the control. (D) Schematic of the experiment. Mixtures of biotin‐labeled NF‐54 and CAF‐54, or biotin‐labeled NF‐54 cells alone prepared as above, were subcutaneously injected in nude mice. Xenografts were resected from the mice 2 days after inoculation, and cells were collected and sorted. Biotin^+^ NFs were sorted by FACS (E, F) and subjected to immunoblot analysis as above (G). (H) Xenografts from D were fixed and subjected to multicolored immunostaining to detect biotin‐labeled NFs (green) and ASPN (red, middle panels) or IL‐1β (red, lower panels). Bottom: Elastica‐Masson trichrome staining. Three mice were examined in each group, and representative images are shown. Scale bar = 50 μm.

First, we demonstrated that CEFs were generated by direct mixture of NFs and CAFs under standard culture conditions. After 48 h of coculture of biotin‐labeled NFs and unlabeled CAFs, cells were collected by FACS with FITC‐streptavidin staining (Fig. 2A,B). Expression of IL‐1β, ASPN, KYNU, and PAPP‐A was increased in biotin‐labeled NFs after coculture with CAFs compared with monocultured NFs (Fig. 2C). In addition, induction of hypoxia‐inducible factor (HIF)‐1α and indoleamine 2, 3‐dioxygenase (IDO)‐1, which catalyzes the initial and rate‐limiting step of the Trp‐Kynurenine (KYN) pathway, was also increased in NFs after coculture with CAFs (Fig. 2C).

Subsequently, a mixture of biotin‐labeled NFs and unlabeled CAFs was subcutaneously injected into nude mice, and xenografts were resected on Day 2 (Fig. 2D). Biotin‐labeled NFs were isolated from the resected explants by FACS (Fig. 2E,F). IL‐1β, ASPN, IDO‐1, KYNU, and PAPP‐A were again upregulated in biotin‐labeled NFs coinjected with CAFs, while these proteins were not detectable or were minimally expressed when NFs alone were injected (Fig. 2G). In addition, immunohistochemical analysis of xenografts further confirmed increased ASPN and IL‐1β expression in biotin‐labeled NFs coinjected with CAFs (Fig. 2H). These results indicated that CAFs educated NFs and generated CEFs in the complete absence of cancer cells *in vivo*.

### 3.3 CEFs promoted cancer cell dispersion and migration via Rac1 activation

**Fig. 3:**
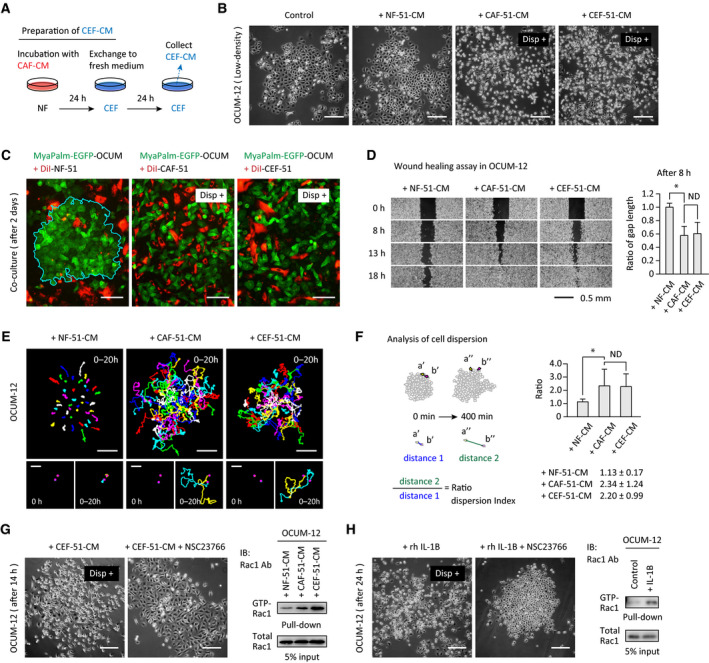
CEFs promoted cancer cell dispersion via Rac1 activation. (A) CEFs were prepared by incubating NFs in a 1 : 1 mixture of fresh complete medium and CAF‐derived conditioned medium (CAF‐CM) for 24 h. The medium was removed, and CEFs were rinsed thoroughly with PBS and maintained in culture for another 24 h in fresh complete medium to collect CEF‐CM. NF‐51, CAF‐51, and CEF‐51 cells were used for all experiments. (B) OCUM‐12 cells were cultured at low density in control fresh medium, or at a 1 : 1 mixture of complete medium and CM derived from NFs, CAFs, or CEFs for 24 h. Disp^+^ indicates cell dispersion. Scale bar = 200 μm. (C) DiI‐labeled fibroblasts (red) and MyrPalm‐EGFP labeled OCUM‐12 cells (green) were mixed (1 : 1), cocultured for 2 days and observed under a confocal microscope. Scale bar = 200 μm. (D) Wound‐healing assay of OCUM‐12 cells incubated with NF‐CM, CAF‐CM, or CEF‐CM. The same fields were monitored. The gap length was measured at 8 h and expressed as the ratio to the cells incubated with NF‐51‐CM. *P < 0.05 (*n* = 4). The data in bar charts are presented as the mean ± SD. (E, F) Tracking of OCUM‐12 cells incubated with each CM. OCUM‐12 cells expressing H2BJ‐EGFP were imaged at 10‐min intervals. Nuclear motion was tracked and drawn as a trajectory I, and used for calculation of neighboring cell distance (F). (E, bottom) Representative tracking images of adjacent neighboring cells, with the initial points of cell tracking marked by magenta dots. Scale bar, 200 µm (upper), 100 µm (bottom). (F) Neighboring cell distances were determined at 400 min and expressed as the ratio to the initial distance. A total of 15 pairs of cells were measured in each group. *P < 0.05. The data in bar charts are presented as the mean ± SD. (G) Left: Suppression of OCUM‐12 dispersion by simultaneous addition of NSC23766 (50 μm, Rac1 inhibitor) with CEF‐CM. Right: Rac1 activity of OCUM‐12 cells treated with each CM for 24 h. Cell lysates were pulled down by GST‐PBD and immunoblotted with anti‐Rac1 to detect GTP‐bound Rac1 (activated Rac1). Scale bar = 200 μm. (H) Left: Dispersion of OCUM‐12 cells with recombinant IL‐1β (20 ng·mL^−1^) with or without NSC23766 (50 μm). Right: OCUM‐12 cells were treated with recombinant IL‐1β for 2 h and subjected to a GTP‐Rac1 pull‐down assay. Scale bar = 200 μm. Statistical significance was calculated using a one‐way ANOVA followed by Tukey's *post hoc* tests (*P < 0.05).

CEFs were prepared by incubating NFs with CAF‐CM (Fig. 3A). To determine the biological effects of soluble factors secreted by CEFs, OCUM‐12 cells were incubated with CMs of NFs, CAFs, or CEFs. Although NF‐CM did not cause significant changes in OCUM‐12 cells, CAF‐CM and CEF‐CM caused dispersion of OCUM‐12 cells (Fig. 3B). Similar results were obtained by CEFs prepared from another patient (Fig. S1A). CM from biotin‐labeled NFs isolated from xenografts formed using the NF/CAF mixture induced a similar dispersion of OCUM‐12 cells (Fig. S1B). In addition, direct coculture of OCUM‐12 cells with CAFs or CEFs, but not with NFs, induced dissociation of the cancer cell nest (Fig. 3C).

A wound‐healing assay was used to confirm the effect of CEF‐CM on cancer cell migration. Restoration of the cell sheet gap was enhanced by incubation with CEF‐CM relative to NF‐CM (Fig. 3D). Subsequently, OCUM‐12 cells were monitored during incubation with NF‐CM, CAF‐CM, or CEF‐CM, and dispersion of OCUM‐12 cell nests was examined using cell tracking in time‐lapse images. The trajectories of OCUM‐12 cells at the end of the incubation period were longer in cells incubated with CEF‐CM than in those incubated with NF‐CM (Fig. 3E, Fig. S1C). Next, adjacent cells at the edges of the nests were tracked, and the distances between the cells were monitored to assess the degree of cell dispersion (Fig. 3F). CEF‐CM or CAF‐CM promoted cell dispersion, as demonstrated by a greater distance between cells (Fig. 3F).

To examine the effects of CEF‐CM on the migration of single cell, a Boyden chamber assay was performed. Migration of OCUM‐12 cells was increased by CAF‐CM and CEF‐CM compared with NF‐CM, suggesting that CEFs can induce the migration of OCUM‐12 (Fig. S1D). To confirm this, the migration velocity of individual cells was analyzed. As shown in Fig. S1E, CEF‐CM also increased the migration velocity of OCUM‐12 cells.

Asporin promotes cell motility by activation of Rac1 via upregulation and association with CD44 [16]. Co‐treatment with CEF‐CM and the Rac1 inhibitor NSC23766 inhibited OCUM‐12 cell dispersion in response to CEF‐CM (Fig. 3G, left). By contrast, co‐treatment with CEF‐CM and Y‐27632 (an inhibitor of the Rho‐associated protein kinase ROCK) or GM6001 (a matrix metalloproteinase inhibitor) did not affect OCUM‐12 dispersion (Fig. S1F). Pull‐down assays using the GTP‐Rac1 binding domain of PAK1 demonstrated that CEF‐CM activated Rac1 (Fig. 3G, right). In addition, we assessed the role of IL‐1β, which is also known to induce Rac1‐mediated cell migration [26]. Recombinant IL‐1β treatment induced OCUM‐12 cell dispersion, which was attenuated by the Rac1 inhibitor NSC23766 (Fig. 3H, left). Rac1 activation by IL‐1β was also confirmed by pull‐down assay (Fig. 3H, right).

By contrast, CEF‐CM did not affect the proliferation of OCUM‐12 cells (Fig. S1G).

### 3.4 CEFs suppressed CD8^+^ T cells via the kynurenine pathway and activated IGF‐I signaling

**Fig. 4:**
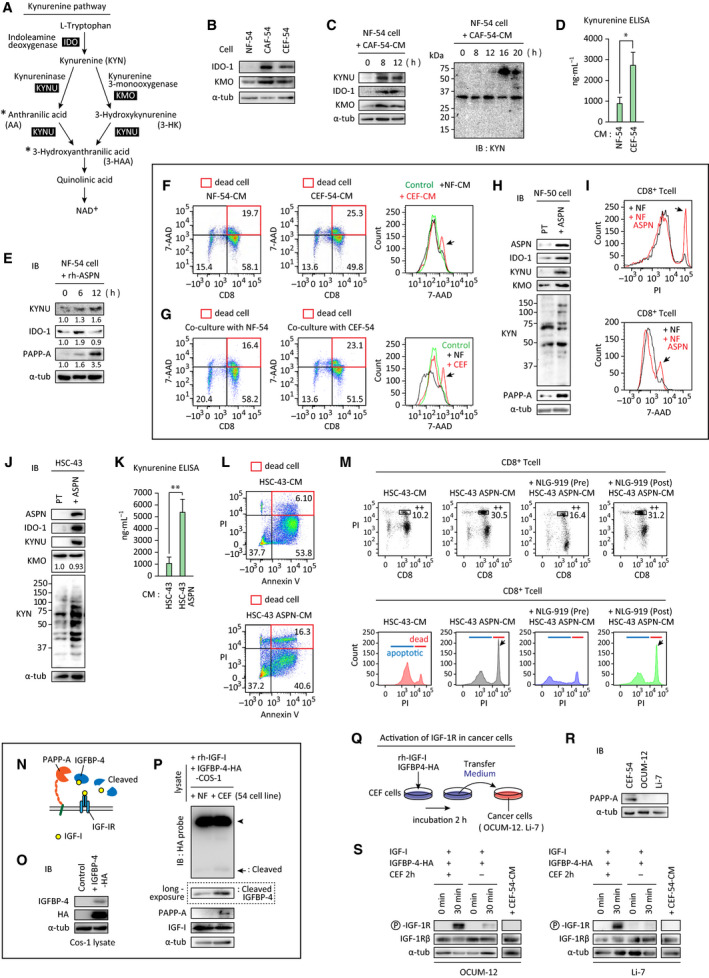
Kynurenine and IGF‐I pathways were activated in CEFs. (A) Simplified schematic of the kynurenine pathway. (B) Cell lysates of NF‐54, CAF‐54, and CEF‐54 were subjected for immunoblot with indicated antibodies. (C) Western blots in NF‐54 cell lysates after treatment with CAF‐CM for indicated period. Right: The bands indicate the protein(s) incorporated KYN. (D) The amount of KYN in each CM was examined by ELISA. *P < 0.05 (*n* = 4. *t*‐test). The data in bar charts are presented as the mean ± SD. (E) NF‐54 cells were incubated with recombinant ASPN (20 ng·mL^−1^). Cell lysates were prepared and subjected to western blot. The band intensities were quantified and normalized to α‐tubulin expression, then, expressed as the relative ratio to the control untreated cells in each panel. (F) CD8^+^ T cells were incubated with NF‐54‐CM or CEF‐54‐CM for 2 days and subjected to FACS analysis. Dot plots of 7‐AAD and CD8 (left), and histogram of 7‐AAD (right). In dot plots, the numbers indicate the percentage of the cells in each fraction. (G) Biotin‐labeled NF‐54 cells were cocultured with CAF‐54 cells for 3 days. Biotin‐labeled NFs were isolated by streptavidin‐coupled microbeads and cocultured with CD8^+^ T cells for 2 days. CD8^+^ T cells were collected, and dead cells (arrow) were detected by 7‐AAD staining. (H–M) ASPN was stably overexpressed in NF‐50 (H, I) or HSC‐43 cells (J–M). (H, J) Western blot analysis of each molecules in cell lysates of parent (pt) cells or ASPN‐expressing cells. The value below the KMO panel indicates the ratio of the band intensity to the control after normalized to α‐tubulin expression. (K) The amount of KYN in each CM was examined by ELISA. **P < 0.01 (*n* = 4. *t*‐test). The data in bar charts are presented as the mean ± SD. (I, L, M) CD8^+^ T cells were incubated with each CM for 2 days and subjected for FACS analysis without fixation. Representative results of three independent assays are shown. Dead cells were evaluated by PI, 7‐AAD, or Annexin‐V staining, and indicated by arrows in the histogram. (M) +NLG‐919 (pre); CM was prepared from HSC‐43 ASPN cells pretreated with an IDO‐1 inhibitor (5 μm). As a control, NLG‐919 was directly added to CD8^+^ T cells together with HSC‐43 ASPN‐CM (+NLG‐919, post). Boxed area indicates dead CD8^+^ T cells. (N) PAPP‐A‐mediated liberation of IGF‐I. (O) Western blot of COS‐1 cells transfected with IGFBP‐4‐HA. (P) *In vitro* cleavage of IGFBP‐4‐HA by cell lysates of NFs or CEFs was examined. Cleaved and uncleaved IGFBP‐4‐HA (arrow and arrowhead, respectively) were detected by immunoblot with anti‐HA antibody. Longer exposure of cleaved IGFBP‐4‐HA is shown at the bottom. (Q) Activation of IGF‐IR signaling in cancer cells by IGF‐I/IGFBP‐4 plus CEF‐54‐CM was examined as described in Section 2.8. (R) Immunoblot of PAPP‐A in CEFs and cancer cells. (S) Activation of IGF‐IR in OCUM‐12 or Li‐7 cells was detected by anti‐phospho‐IGF‐IRβ antibody. CEF(−) indicates that the IGF‐I/IGFBP‐4 mixture was directly added to cancer cells. Right panels (+CEF‐CM): Activation of IGF‐IR was not induced by CEF‐CM alone.

We next focused on KYNU and pregnancy‐associated plasma protein‐A (PAPP‐A), which were among the most highly increased genes in CEFs relative to NFs or CAFs (Fig. 1G). KYNU is involved in downstream tryptophan (Trp) metabolism (Fig. 4A asterisk). Additionally, IDO‐1 and kynurenine 3‐monooxygenase (KMO), upstream enzymes in Trp metabolism, were also increased in CEFs (Fig. 4B,C left). The metabolites of these enzymes, kynurenine (KYN) and 3‐hydroxyanthranlic acid (3‐HAA), inhibit T‐cell function [27]. In addition, increased KYN in CEFs was observed by immunoblot analysis (Fig. 4C, right) and ELISA (Fig. 4D). PAPP‐A activates IGF‐I/IGF‐I receptor signaling. Interestingly, expressions of KYNU, IDO‐1, and PAPP‐A were all increased by treatment of NFs with recombinant ASPN (Fig. 4E), suggesting that ASPN induces expression of these proteins in CEFs.

The immunosuppressive effects of CEFs were examined. When CD8^+^ T cells isolated from mouse spleens were treated with NF‐CM or CEF‐CM, CEF‐CM increased T‐cell death (Fig. 4F, Fig. S2A). The cytocidal effect of CEF on CD8^+^ T cells was slightly higher when CEFs were directly cocultured with CD8^+^ T cells (Fig. 4G, Fig. S2B). Because ASPN induced expression of KYNU and IDO‐1, we further examined T‐cell suppression by ASPN‐overexpressing NFs (NF‐ASPN). NF‐ASPN induced expression of IDO‐1, KYNU, KMO, and the production of KYN (Fig. 4H). Cytofluorometric analysis with PI or 7‐aminoactinomycin D (7‐AAD) revealed that NF‐ASPN increased the death of CD8^+^ T cells relative to parental NFs (Fig. 4I).

The viability of NFs gradually decreased after gene introduction, and therefore, it remained possible that the above effects were due to NF death after ASPN overexpression. To confirm ASPN‐mediated upregulation of the KYN pathway in other cells, and to confirm that its cytocidal effect on CD8^+^ T cells depends on KYN pathway metabolites, we used a gastric cancer cell line, HSC‐43, which is more stable than NF cells after gene overexpression. When ASPN was expressed in HSC‐43 cells, which does not express ASPN endogenously, expression of IDO‐1, KYNU, and KYN was also increased (Fig. 4J). This effect was abrogated by knockdown of ASPN with siRNA (Fig. S2C). In HSC‐43 cells, KMO expression was not affected by ASPN (Fig. 4J). The increase in KYN secretion in HSC‐43 ASPN cells was confirmed by ELISA (Fig. 4K). Cytofluorometric analysis indicated that Annexin‐V^+^/PI^+^ dead CD8^+^ T cells were markedly increased by HSC‐43 ASPN‐CM (Fig. 4L). The cytocidal effect on CD8^+^ T cells was blocked by pretreating HSC‐43 ASPN cells with an IDO‐1 inhibitor, while direct addition of the IDO‐1 inhibitor to CD8^+^ T cells treated with HSC‐43 ASPN‐CM did not affect cell death (+NLG‐919; pre and post, respectively; Fig. 4M). These results indicate that ASPN activated secretion of Trp‐KYN metabolites to attenuate T‐cell function.

We next examined the effect of CEFs on IGF‐I signaling. Although the majority of IGF‐I is bound to IGF binding protein (IGFBP), which renders IGF‐I unable to bind to IGF‐IR, PAPP‐A cleaves IGFBP‐4 bound to IGF‐I, liberating the IGF‐I [28]. When recombinant IGF‐I was mixed with a protein lysate containing IGFBP‐4 and further incubated with lysates of CEFs or NFs, IGFBP‐4 cleavage was only inducted by the CEF lysate, which contained PAPP‐A (Fig. 4N–P). CEF activation of IGF‐IR in cancer cells was also examined (Fig. 4Q). When OCUM‐12 cells or Li‐7 cells, which do not express PAPP‐A (Fig. 4R), were incubated with medium containing recombinant IGF‐I and IGFBP‐4, IGF‐IR was minimally activated, if at all [Fig. 4S, CEF (−)]. Co‐treatment with the mixture of IGF‐I, IGFBP‐4, and CEF‐CM caused phosphorylation of IGF‐IRβ, suggesting that CEF‐mediated liberation of IGF‐I induced receptor activation (Fig. 4S).

### 3.5 CEFs localize to the peripheral invading region of gastric cancer

To estimate the size and location of CEFs in tumors, we focused on the genes most highly upregulated in CEFs relative to NFs or CAFs, including KYNU, PAPP‐A, and CXCL6. Immunohistochemical screening of human gastric cancer specimens revealed that CXCL6 was frequently expressed in cancer cells at a high level, while staining in fibroblastic cells was observed in limited cases (Fig. S2D). On the other hand, KYNU and IDO‐1 were primarily detected in stromal cells. In addition, CM derived from two gastric cancer cell lines did not induce KYNU or IDO‐1 in NFs, while PAPP‐A was slightly upregulated by CM from one of the two cell lines (Fig. S2E). Taken together with the observation that KYNU and its upstream mediator IDO‐1 were upregulated by ASPN, ASPN^high^/IDO‐1^high^/KYNU^high^/α‐SMA^−^ fibroblasts were considered to represent CEFs and were further examined.

**Fig. 5:**
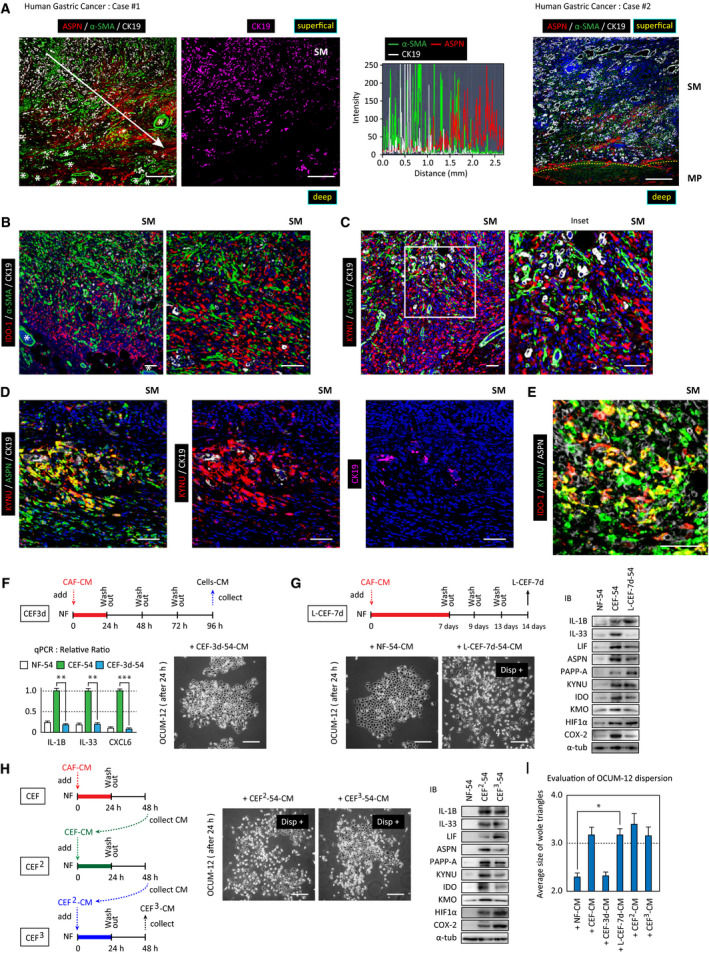
Localization of CEFs in gastric cancer and continuous generation of CEFs. (A) Paraffin sections of human gastric cancer specimens (case #1 and case #2) were immunostained with anti‐ASPN (red), anti‐α‐SMA (green), and anti‐Cytokeratin19 (white or magenta). Asterisks indicate blood vessels. Scale bar = 500 μm. The distribution of positive cells by each antibody was quantified using zen software. The fluorescence intensity was measured in the direction of invasion (white arrow) and plotted from proximal to distal (right panel). Dotted line indicates border between SM: submucosa and MP: muscle layer. In both cases, α‐SMA was stained in muscle layer. (B–E) Gastric cancer specimens (case#1) were immunostained with indicated antibodies. Confocal microscope images of similar regions in submucosa (SM) were shown. Scale bar = 50 μm. (F) Top: CEF‐3d indicates CEFs 3 days after generation. Bottom, left: mRNA levels of IL‐1β, IL‐33, and CXCL6 in CEFs 3 days after preparation (CEF‐3d) were compared with NFs by qRT‐PCR (*n* = 3, **P < 0.01 and ***P < 0.001). Right: CM of CEF‐3d did not disperse OCUM‐12 cells. The data in bar charts are presented as the mean ± SD. (G) L‐CEF: NF‐54 cells were treated with CAF‐CM for 7 days. L‐CEF‐7d indicates L‐CEFs 7 days after removal of CAF‐CM. Left; Representative image of OCUM‐12 cells incubated with L‐CEF‐7d‐CM. Right; Expression of CEF marker genes in L‐CEF‐7d was examined by western blot. (H) CEF^2^ and CEF^3^ (CEF‐educated fibroblasts): the second and the third generation of CEFs were prepared as indicated in the scheme. OCUM‐12 cells incubated with the NF‐CM, CEF^2^‐CM, or CEF^3^‐CM for 24 h. Representative images are shown. Disp^+^ indicates cell dispersion. Scale bars = 200 μm. Right: Immunoblot analysis of CEF marker genes in CEF^2^ and CEF^3^ cells with indicated antibodies. (I) Cell dispersion was analyzed using Delaunay triangulation plot. The graph indicates the average size of whole triangles (*P < 0.05). Statistical significance was calculated using a one‐way ANOVA followed by Tukey's *post hoc* tests. The data in bar charts are presented as the mean ± SD.

First, expressions of ASPN and α‐SMA were compared. While α‐SMA^+^ CAFs were observed at the highest density in the cancer core region, where cancer cells compacted, ASPN was mainly expressed in the distal area (Fig. 5A, Fig. S3A). In the adjacent areas, α‐SMA and ASPN expression did not apparently overlap (Fig. S3B).

Next, the localization of IDO‐1 and KYNU was compared with that of α‐SMA in the same specimens. IDO‐1 and KYNU expression did not overlap with α‐SMA^+^ CAFs, and they were also mainly detected in the stromal cells, especially at the invading edge of the tumor periphery (Fig. 5B,C, Fig. S3C,D). In stromal tissue, ASPN was solely detected in vimentin^+^ fibroblasts, and many vimentin^+^ fibroblasts were also KYNU^+^ or IDO‐1^+^ (Fig. S3E).

ASPN^high^/ KYNU^high^ fibroblasts were detected in the tumor periphery, where few cancer cells reside (Fig. 5D), and many were also IDO‐1‐positive (Fig. 5E) and KMO‐positive (Fig. S3F). Taken together, these data indicate that ASPN^high^/IDO‐1^high^/KYNU^high^/α‐SMA^−^ fibroblasts, which represent CEFs, are mainly located at the peripheral invading region of the tumor.

### 3.6 CEFs were sequentially generated

To understand how the CEF phenotype was maintained or reversed, we subsequently examined the stability of the CEF phenotype. When CEFs were prepared by 24 h incubation with CAF‐CM as above, expression of *IL‐1B*, *IL‐33,* and *CXCL6* in CEFs returned to basal levels 72 h after removal of the CAF‐CM (CEF‐3d) (Fig. 5F), and CM collected from these CEFs (CEF‐3d‐CM) did not induce OCUM‐12 cell dispersion (Fig. 5F,I, Fig. S4A). These results indicated that induction of the CEF phenotype by short‐term exposure to CAF‐CM was transient. On the other hand, when CEFs were prepared by 7‐day incubation with CAF‐CM, many genes upregulated in CEFs by this long‐term exposure to CAF‐CM were continuously expressed for up to 7 days after removal of the CAF‐CM (L‐CEF‐7d; Fig. 5G). Furthermore, CM collected from these CEFs (L‐CEF‐7d‐CM) induced OCUM‐12 cell dispersion (Fig. 5G,I, Fig. S4A,B). Therefore, CEFs could revert to an NF phenotype after short‐term CAF education, but the stability of the CEF phenotype depended on the duration of CAF education.

Next, we examined whether CEFs sequentially educated NFs. When NFs were incubated with CEF‐CM, the resulting CEF‐educated fibroblasts (CEF^2^) also secreted factors that promoted OCUM‐12 cell dispersion (Fig. 5H,I, Fig. S4A). Repeating this cycle revealed that the effect of CEF education was successively transmitted for at least three generations (CEF^3^; Fig. 5H). Expression of inflammatory cytokines, ASPN, KYNU/ IDO‐1, and PAPP‐A, was maintained in CEF^2^ and CEF^3^ cells (Fig. 5H, right). These results suggest that CEFs successively activated NFs to promote tumor expansion.

### 3.7 CEFs were induced by ROS‐mediated activation of NF‐κB and HIF‐1α

**Fig. 6:**
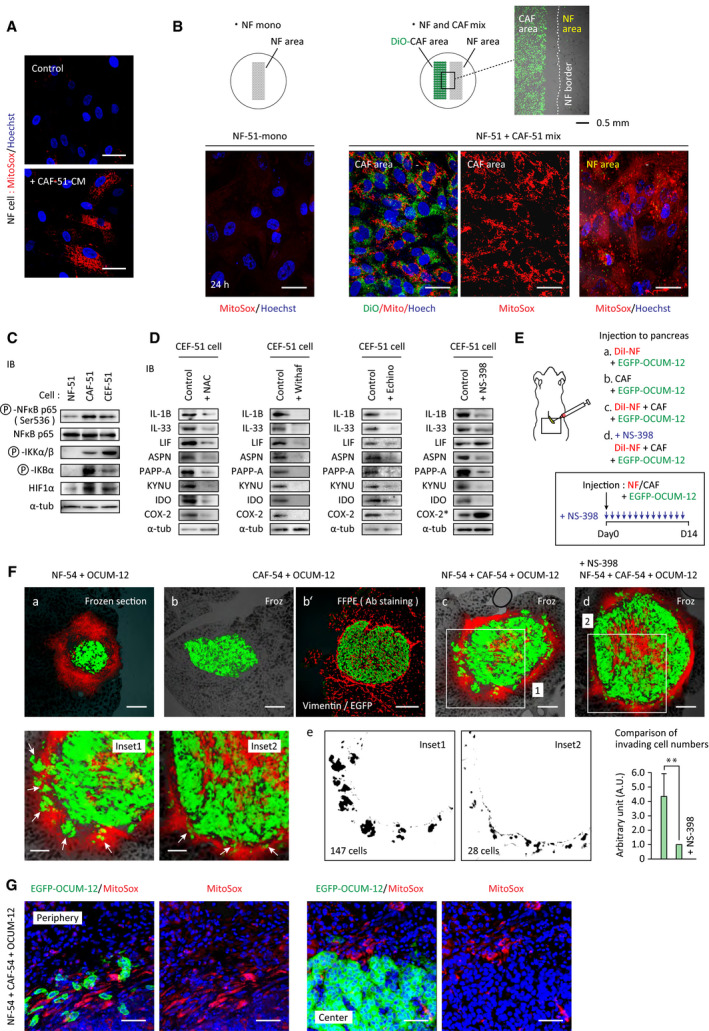
CEFs are induced by ROS generation and facilitate tumor dissemination. (A) NFs were treated with or without CAF‐CM for 20 h and stained with MitoSox (red) and Hoechst (blue). Scale bar = 30 μm. (B) Two precisely defined patches of NFs and DiO‐labeled CAFs (green) separated by a 500 μm gap were prepared using culture inserts (ibidi). After incubation for 24 h, cells were stained with MitoSox and Hoechst. Scale bar = 30 μm. (C) Expression and activation of NF‐κB pathway and HIF‐1α in NFs, CAFs, and CEFs was examined by immunoblotting with the indicated antibodies. (D) NFs were treated with CAF‐CM with or without NAC (3 mm, ROS scavenger), Withaferin A (2 μm, NF‐κB signaling inhibitor), Echinomycin (0.5 μm, HIF‐1α inhibitor), or NS‐398 (10 μm, COX2 inhibitor) for 24 h. Expression levels of CEF markers were examined by western blot as indicated. *COX2 expression is known to be upregulated by NS‐398 as a feedback mechanism [52]. In A–D, NF‐51, CAF‐51, and CEF‐51 cells were used. (E, F) Tumor dissemination model in the murine pancreas. EGFP‐expressing OCUM‐12 cells (1 × 10^5^) were transplanted in mouse pancreases together with either DiI‐labeled NF‐54 (3 × 10^5^, a), unlabeled CAF‐54 (2 × 10^5^, b, b′), or a mixture of DiI‐NF‐54 and CAF‐54 cells (c, d). NS‐398 was injected daily in the peritoneal cavity (5 mg·kg^−1^·day^−1^, d). Mice were sacrificed at Day 14, and xenografts were frozen‐sectioned (a, b, c, d), or formalin‐fixed and paraffin‐embedded (FFPE) and subjected for immunostaining with anti‐Vimentin antibody to detect fibroblasts (b′). Maximum cut surface was shown in each xenograft. Boxed areas are enlarged at the bottom. Arrows indicate cancer cell invasion into peripheral fibroblasts. Five mice were examined in each group, and representative images are shown. (e) Areas of cancer cell peripheral invasion were marked (black), and the cells in marked area were counted in whole circumference of the tumor. Examples are shown. Scale bars = 200 μm (a–d), 100 μm (insets). **P < 0.01 (*t*‐test). The data in bar charts are presented as the mean ± SD. (G) Mixture of EGFP‐expressing OCUM‐12 cells and unlabeled NFs and CAFs was injected in mice pancreas as above. Xenografts were excised, dissected by vibratome (200 μm), and immediately stained with MitoSox (red). The tissues were fixed and frozen‐sectioned for confocal microscope imaging. Scale bar = 50 μm.

We further examined the mechanism of CAF‐mediated education of NFs. Several factors are known to cause transcriptional reprogramming, including oxidative stress through increased ROS generation, hypoxic changes, and lactate uptake [29, 30, 31]. Staining with MitoSox, a superoxide indicator, revealed that ROS generation was increased in NFs treated with CAF‐CM (Fig. 6A). Coculture of NFs and CAFs induced a similar response. When NFs and CAFs were cultured in separated areas of the same dish, high ROS levels were induced in NFs (Fig. 6B). These results suggest that CAF‐mediated ROS production in NFs contributed to NF reprogramming and acquisition of the CEF phenotype.

Oxidative signaling sequentially activates NF‐κB and HIF‐1α, which coordinately regulate inflammatory cytokines [32]. Activation of NF‐κB signaling, including phosphorylation of NF‐κB p65, IκB kinase (IKK), and inhibitor of NF‐κB (IKB), occurred in CEFs, which was accompanied by increased HIF‐1α levels (Fig. 6C). Repression of ROS levels in CEFs by addition of a ROS scavenger, inhibition of NF‐κB (Withaferin A), HIF‐1α (Echinomycin), or COX2 (NS‐398) attenuated expression of most genes upregulated in CEFs (Fig. 6D). These results suggested that the CEF phenotype depended primarily upon ROS‐mediated activation of NF‐κB/HIF‐1α signaling.

### 3.8 CEFs promoted tumor dissemination *in vivo*

To assess the functional significance of CEFs on tumor spreading *in vivo*, gastric cancer cells were mixed with NFs or CEFs and injected into nude mice stomach. A cancer cell line that did not directly upregulate CEF markers in NFs was selected for these experiments. OCUM‐12‐CM did not induce ASPN, inflammatory cytokines, HIF‐1α, IDO‐1, or KYNU expression in NFs (Fig. S2E) *in vitro*, suggesting changes to the tumor would reflect the effects of the coinjected CEFs.

Invasion of OCUM‐12 cells was aggravated by CEFs. By the mixture with NFs, OCUM‐12 cells expanded by small clusters (Fig. S5a) and remained in submucosal layer (Fig. S5c,e,g,i). On the other hand, OCUM‐12 cells were invaded by scattered pattern with inflammatory responses in the stroma (Fig. S5b) and entered into muscle layer by mixture with CEFs (Fig. S5d,f,h,j).

Next, cancer cells were mixed with NFs and/or CAFs and injected into the pancreases of nude mice, which generated CEFs *in situ*, and mimicked the dissemination of gastric cancer cells in adjacent organs (Fig. 6E). As a control, mice were inoculated with the same number of cancer cells mixed with either CAFs or NFs alone.

The area of cancer cells was increased by coinjection of OCUM‐12 cells with CAFs and NFs relative to CAFs or NFs alone (Fig. 6Fa–c). Dissemination of OCUM‐12 cells into the peripheral fibroblasts was evident in mice coinjected with a mixture of cancer cells, CAFs, and NFs (Fig. 6Fc, Fig. S6A). A COX2 inhibitor did not reduce the tumor size, although peripheral cancer cell invasion was effectively blocked (Fig. 6Fd,e). In addition, when the amount of ROS in the tumor was evaluated by MitoSox labeling, higher ROS generation was detected in the fibroblasts (Fig. 6G). These results suggested that generation of CEFs *in vivo* promoted invasion and dissemination of cancer cells in this model.

When induction of KYNU expression in fibroblasts was examined, it was rarely detected in xenografts coinjected with CAFs alone, while many fibroblasts expressed KYNU after coinjection with human CAFs and NFs (Fig. S6B). These results suggest that the effect of human CAFs on mouse resident fibroblasts was rather weak in this model.

## 4 Discussion

In the present study, we propose CEFs as a novel type of pro‐tumor fibroblasts generated by CAF education of NFs, in the absence of direct interaction with cancer cells. CEFs are pro‐inflammatory fibroblasts that can induce T‐cell death and IGF‐I signaling in tumors, and they were generated not only *in vitro* but also *in vivo*.

CEFs are generated via education by CAFs or other CEFs. Because ASPN is predominantly expressed in tumor fibroblasts, but rarely expressed in gastric cancer cells (around 10%) [33], and soluble ASPN upregulated unique pathways in NFs, ASPN may be important as an educational factor, and the genes upregulated by soluble ASPN could represent the CEF phenotype. Recombinant ASPN induced expression of KYNU and IDO‐1 within several hours in NFs, while they were not induced by CM derived from at least other two cancer cells. Therefore, we selected tumor fibroblasts with molecular features of ASPN^high^/IDO‐1^high^/KYNU^high^/α‐SMA^−^ as CEFs. Although we cannot say at present that CEFs are never induced by cancer cells, as it is impossible to test every cancer cell lines, these cells mainly represent CEFs. On the other hand, some lung cancer cells also induce tryptophan 2, 3‐dioxygenase (TDO2; another rate‐limiting enzyme in Trp‐catabolism)‐positive myofibroblasts adjacent to the cancer region [34]. The ASPN‐KYN pathway identified here activated nonmyogenic fibroblasts that are distinct from these and are located in a distal region of the tumor.

**Fig. 7:**
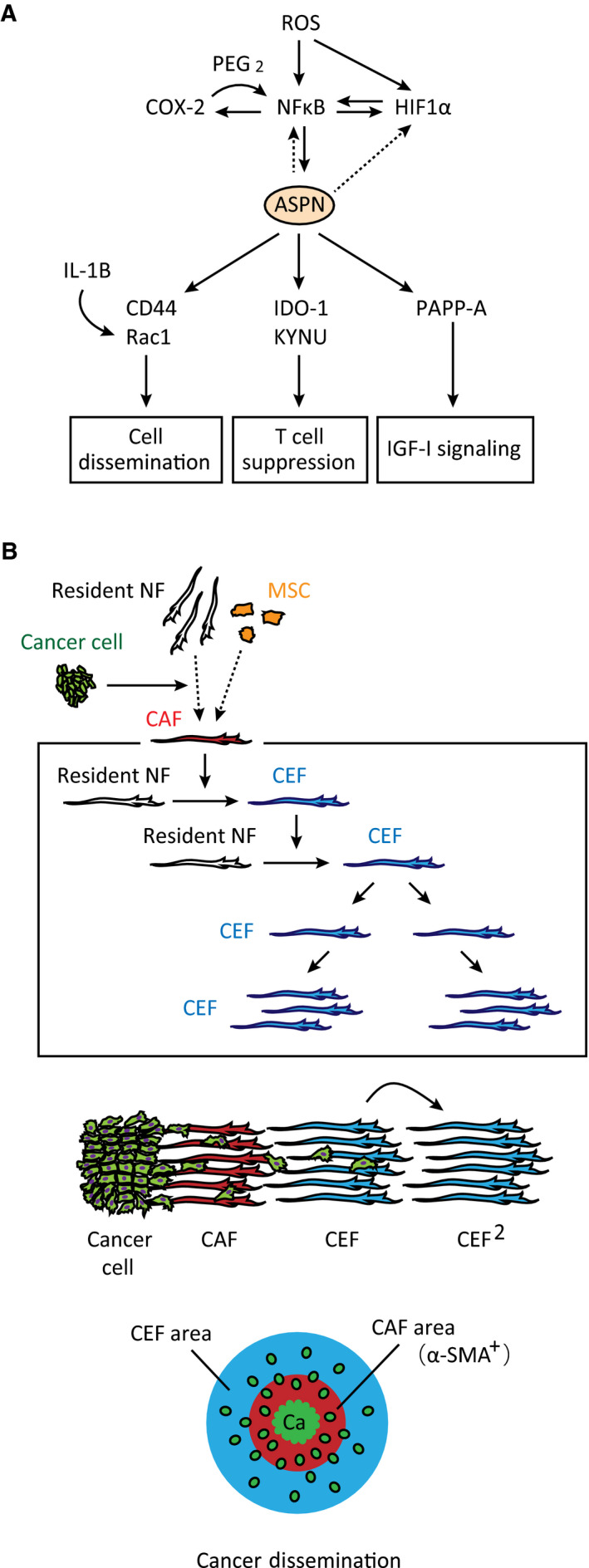
Model of sustained expansion of pro‐tumor fibroblasts by CEFs. (A) CAFs induce ROS production in NFs, which activates the NF‐κB pathway. NF‐κB upregulates HIF‐1α and COX2, which mutually activate one another in a positive feedback loop. ASPN is transcriptionally upregulated by NF‐κB and promotes CD44/Rac1‐mediated cancer cell dissemination, suppresses CD8^+^ T cell through kynurenine pathway, and activates IGF‐I signaling via PAPP‐A. CEF‐secreted IL‐1β also activates Rac1‐dependent cancer cell dissemination. (B) Model of CEF‐mediated cancer cell dissemination. CAF is generated through the education of resident NFs or bone marrow mesenchymal stem cells by cancer cells. CEFs (blue) sequentially educate resident NFs to further amplify pro‐tumor fibroblasts in the TME, which expands tumor and triggers cancer cell dissemination.

We observed that CAFs increased ROS levels in NFs. ROS upregulates HIF‐1α and COX2 via stimulation of NF‐κB transcriptional activity and by inhibition of HIF‐1α ubiquitination and degradation [35, 36]. Because HIF‐1α and NF‐κB mutually activate one another, the inflammatory response is amplified by a positive feedback loop [37, 38, 39]. In addition, COX2‐mediated synthesis of prostaglandin E2 (PGE2) also amplifies NF‐κB signaling [40]. As a downstream target of NF‐κB signaling, ASPN is upregulated because its promoter contains a binding site for NF‐κB p65 [41]. In turn, ASPN further increases NF‐κB and HIF‐1α expression [33] (Fig. 7A). Therefore, ROS production is a major trigger of the inflammatory response in CEFs.

Although TGF‐β signaling is activated in some CAFs, TGF‐β transcription and activation/expression of SMAD2, p38, and Snail were not elevated in CEFs (Fig. S7A,B). In addition, treatment of NFs with TGF‐β did not induce expression of the CEF marker genes, KYNU and PAPP‐A (Fig. S7C). Taken together, these data suggest that the features of CEFs evaluated in this study were unlikely to have been induced by TGF‐β.

CEFs induced cancer cell spreading via IL‐1β secretion, which activates Rac1 in cancer cells. Moreover, ASPN upregulates CD44 expression and activates Rac1 via interaction with CD44 on the cell membrane [16, 33], which also increases cancer cell spreading (Fig. 7A). In addition, CD44‐mediated upregulation of KYNU also activates cell invasion [42]. On the other hand, epithelial–mesenchymal transition (EMT)‐related genes were not upregulated in cancer cells by CEF‐CM (Fig. S7D). Therefore, dispersion of OCUM‐12 cells by CEF‐CM was due to the activation of cell migration rather than EMT. Because ASPN^high^/IDO‐1^high^/KYNU^high^/α‐SMA^−^ fibroblasts were located in the peripheral invading region, these CEFs may contribute to the peripheral expansion of tumors, as observed in the mouse model (Fig. 7B), and may stimulate cancer cell metastasis.

We also examined whether CEFs activate CAFs by incubating CAFs with CEF‐CM (Fig. S7E). IDO‐1, KYNU, PAPP‐A, and IL‐1B were not upregulated in CEF‐CM‐treated CAFs, suggesting that CEFs do not amplify pre‐existing CAF function, but that newly differentiated CEFs can promote tumor progression. However, further investigation of the feedback effects of CEFs on CAFs is necessary.

The tryptophan‐kynurenine (KYN) pathway suppresses T cells by several mechanisms including depletion of tryptophan and generation of KYN [43]. Furthermore, 3‐hydroxyanthranlic acid (3‐HAA) causes T‐cell apoptosis [44, 45, 46]. Cytocidal effects on CD8^+^ T cell were induced by CEF‐CM and ASPN‐overexpressing cells and were attenuated by blocking the secretion of KYN metabolites into the CM with an IDO‐1 inhibitor. While KYNU catabolizes and reduces the immunosuppressive effects of KYN, the downstream product 3‐HAA suppresses anti‐tumor immunity and correlates with poor survival in lung cancer and highly metastatic breast cancer [47, 48, 49].

In addition, upregulation of KYNU in CEFs did not simply eliminate KYN, but rather increased KYN (Fig. 4C,D), likely via the simultaneous overexpression of IDO‐1. We observed that CD8^+^ T cells were absent from the region enriched in ASPN^high^/KYNU^high^ stromal cells in human gastric cancer specimens, whereas CD8^+^ T‐cell infiltration was observed in the region containing KYNU^low^ stromal cells (Fig. S7F). Because the cytocidal effect of 3‐HAA on T cells is more prominent than that of KYN [44], and higher expression of KYNU in gastric cancer correlates with poorer prognosis (The Human Protein Atlas), comprehensive quantification of Trp‐KYN metabolites in the CEF supernatant, for example, by liquid chromatography‐mass spectrometry, should be performed in future studies.

It has been suggested that CEFs promote the release of IGF‐I via PAPP‐A, which cleaves the complex of IGF‐I and IGFBP‐4 *in vitro* [50] and activates IGF‐I receptor‐mediated signaling in cancer cells. Although IGF‐I expression was not observed in CEFs or the gastric cancer cell lines used in the present study, IGF‐I is known to be expressed in gastric cancer tissues and correlates with poor prognosis [51]. Further large‐scale studies should examine IGF‐I, IGFBP‐4, and PAPP‐A using immunohistology. In the present study, PAPP‐A staining was observed in the stromal fibroblastic cells in only a few of 10 gastric cancer cases examined (Fig. S8A). In such cases, PAPP‐A was detected in the periphery region of the tumor and also at the invading front, where only a few cancer cells infiltrated (Fig. S8B). We also observed many stromal cells co‐expressing PAPP‐A and ASPN (Fig. S8C). These observations may indicate the localization of CEFs; however, this must be confirmed with a larger number of PAPP‐A‐positive human gastric cancers specimens. Taken together, ASPN may function as a key molecule in CEFs that influences cancer cell dissemination, suppression of CD8^+^ T cells, and regulation of IGF‐I signaling (Fig. 7A).

The mechanism underlying the higher expression of KYNU and PAPP‐A in CEFs compared with CAFs, despite the fact that both cell types express ASPN, is not clear at present. ASPN secreted by CEF may act through an autocrine mechanism; alternatively, intracellular ASPN binds to certain transcription factors, which may also regulate gene expression. Therefore, intracellular signaling by ASPN may be regulated by such cell type‐specific modulators, resulting in the preferential induction of KYNU and PAPP‐A in CEFs. In the future, it will be necessary to identify the ASPN modulators involved in the induction of KYNU and PAPP‐A in CEFs.

## 5 Conclusions

Fibroblasts in the TME appear to be educated through diverse mechanisms. CEF production continuously generates TME fibroblasts that promote cancer cell invasion (Fig. 7B). In human tumors, both cancer cells and CAFs are thought to educate neighboring cells; for example, pro‐inflammatory genes are also upregulated in fibroblasts educated by some cancer cells [13]. The CEF ratio may depend on the cancer cell density and the distance of the fibroblasts from the cancer cells. In some gastric cancer specimens, a small number of cancer cells are scattered within a large fibrotic stroma, especially in the tumor periphery (Fig. 5D). Even in portions of the TME without cancer cells, CAF education of NFs could potentially expand pro‐tumor fibroblasts. Moreover, if CEFs have high plasticity and convert to NFs or typical CAFs (e.g., α‐SMA^+^/FAP^+^/PDPN^+^) in some contexts, then CEFs could be a potential target for preventing the expansion of heterogeneous CAFs. Our findings suggest that blocking CEF production may be a promising therapeutic strategy for targeting tumor invasion and dissemination.

## Conflict of interest

The authors declare no conflict of interest.

## Author contributions

MT designed and supervised the experiments. GI gave the first idea and was a major contributor in writing the manuscript. GI, KT, YF, MU, AG, KY, and MY performed part of the experiments. YF and SK analyzed the data with assistance from GI. All authors read and approved the final manuscript.

### Peer review

The peer review history for this article is available at https://publons.com/publon/10.1002/1878‐0261.13077.

## Supporting information


**Fig. S1.** CEFs promoted cancer cell dispersion.

**Fig. S2.** Activation of the KYNU pathway by ASPN, and the effects of cancer cell CM on NFs.

**Fig. S3.** Localization of CEFs in gastric cancer.

**Fig. S4.** Evaluation of cancer cells dispersion by Delaunay triangulation plots.

**Fig. S5.** Tumor dissemination in the murine stomach.

**Fig. S6.** CEFs promote cancer cell invasion *in vivo*.

**Fig. S7.** Evaluation of TGF‐β signaling in CEFs, and infiltration of CD8^+^ T cells in gastric cancer.

**Fig. S8.** Immunohistochemical analysis of PAPP‐A in gastric cancer.

**Table S1.** Primer sequences used for quantitative PCR.

Supplementary Material

## Data Availability

All data related to this study are available from the corresponding author upon reasonable request.
