# Supplementary figures and images for: Cancer‐associated fibroblasts educate normal fibroblasts to facilitate cancer cell spreading and T‐cell suppression
Source: Mol Oncol. 2021 Nov 5;16(1):166–87. doi: 10.1002/1878-0261.13077 (PMC8732346; doi:10.1002/1878-0261.13077)

Supplementary Figure. 1

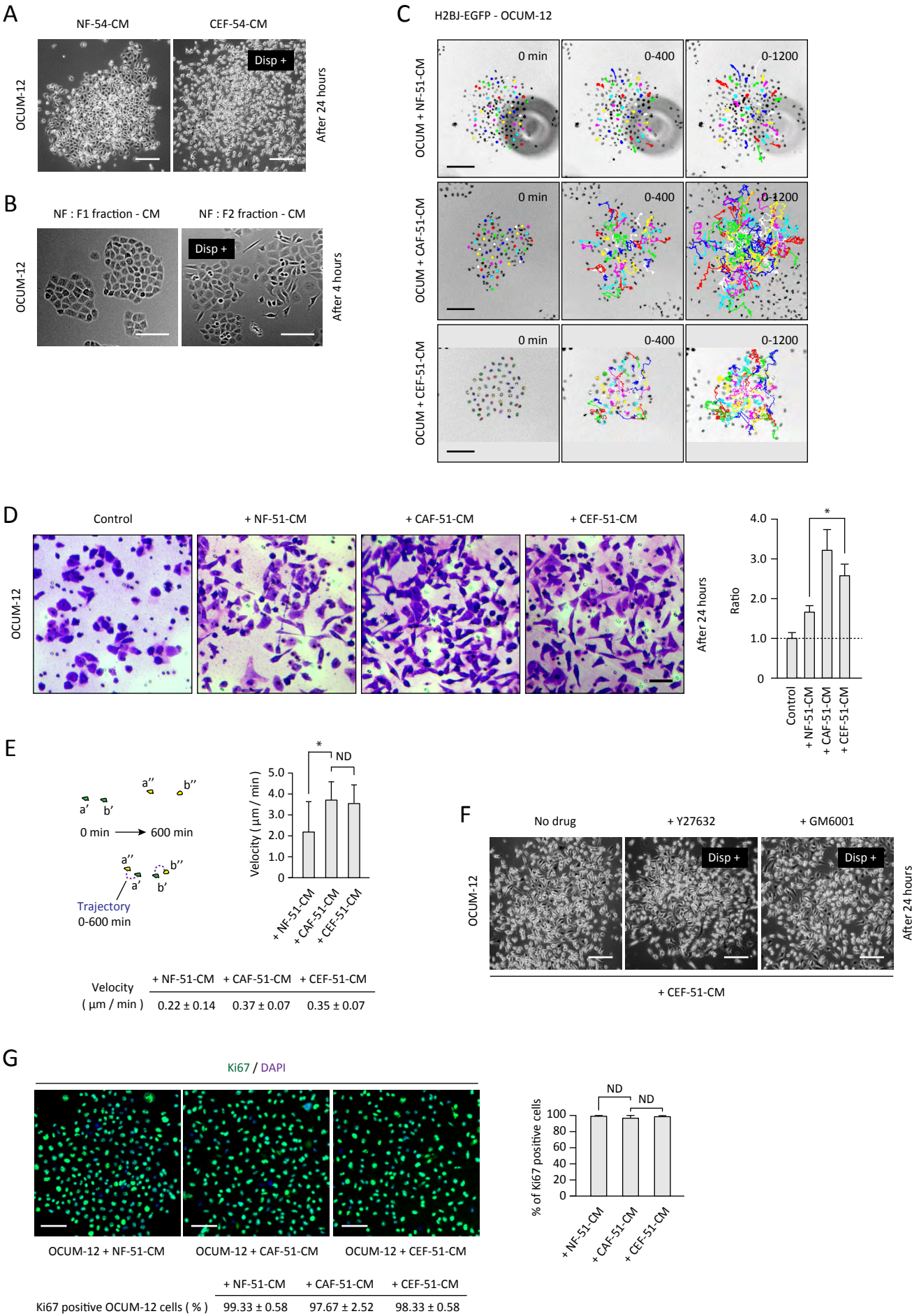

Supplement: Supplementary file 1 — Fig. S1. CEFs promoted cancer cell dispersion. [file MOL2-16-166-s001.pdf]

Supplementary Figure. 2

A

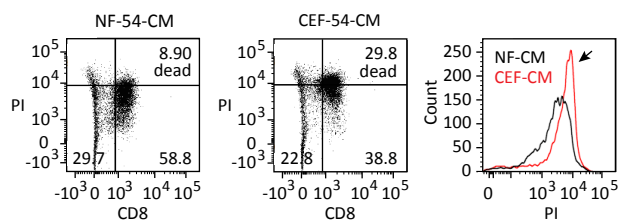

B

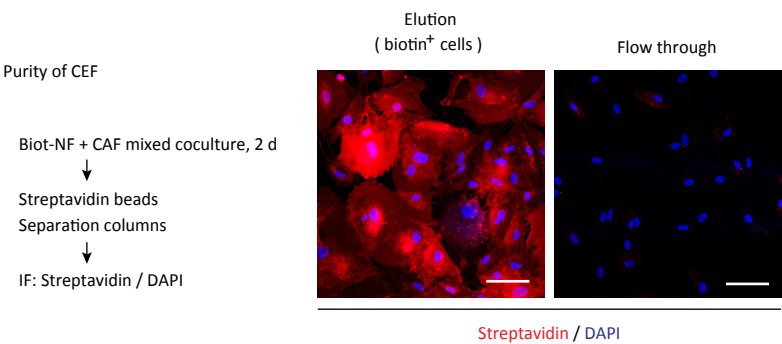

C

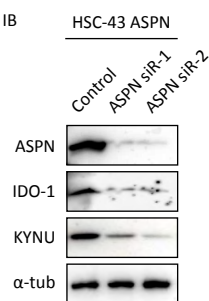

D

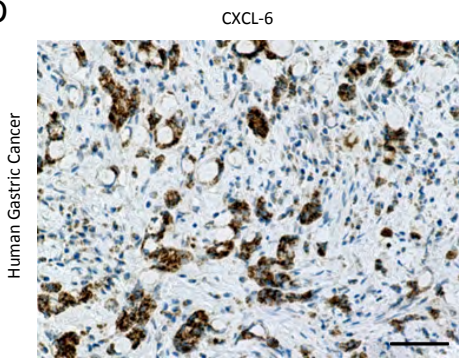

E

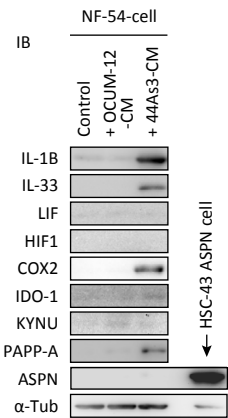

Supplement: Supplementary file 2 — Fig. S2. Activation of the KYNU pathway by ASPN, and the effects of cancer cell CM on NFs. [file MOL2-16-166-s009.pdf]

Supplementary Figure. 4

A Construction of Delaunay triangulation

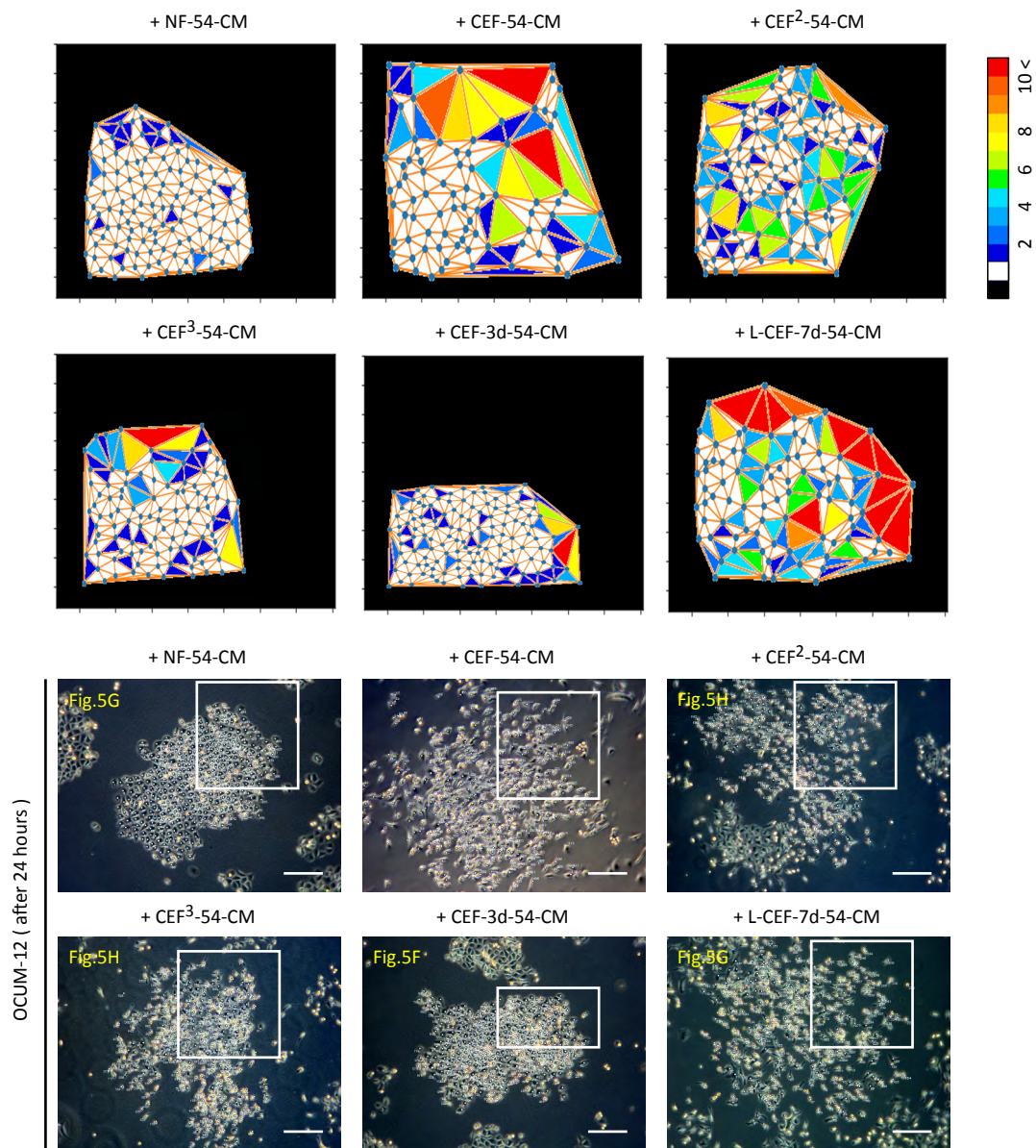

B

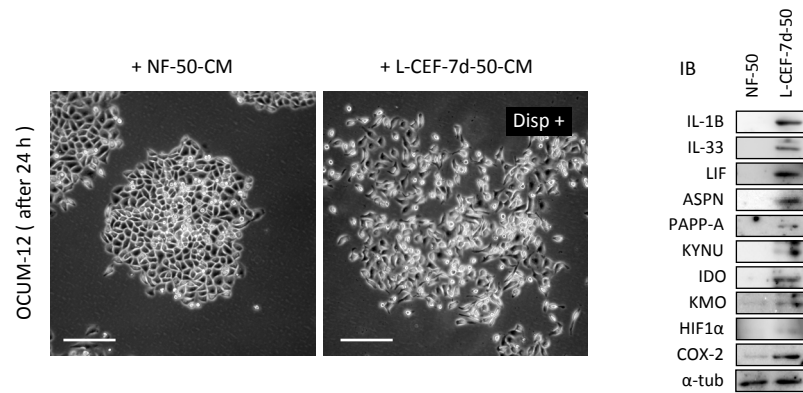

Supplement: Supplementary file 4 — Fig. S4. Evaluation of cancer cells dispersion by Delaunay triangulation plots. [file MOL2-16-166-s004.pdf]

Supplementary Figure. 5

Mice gastric wall

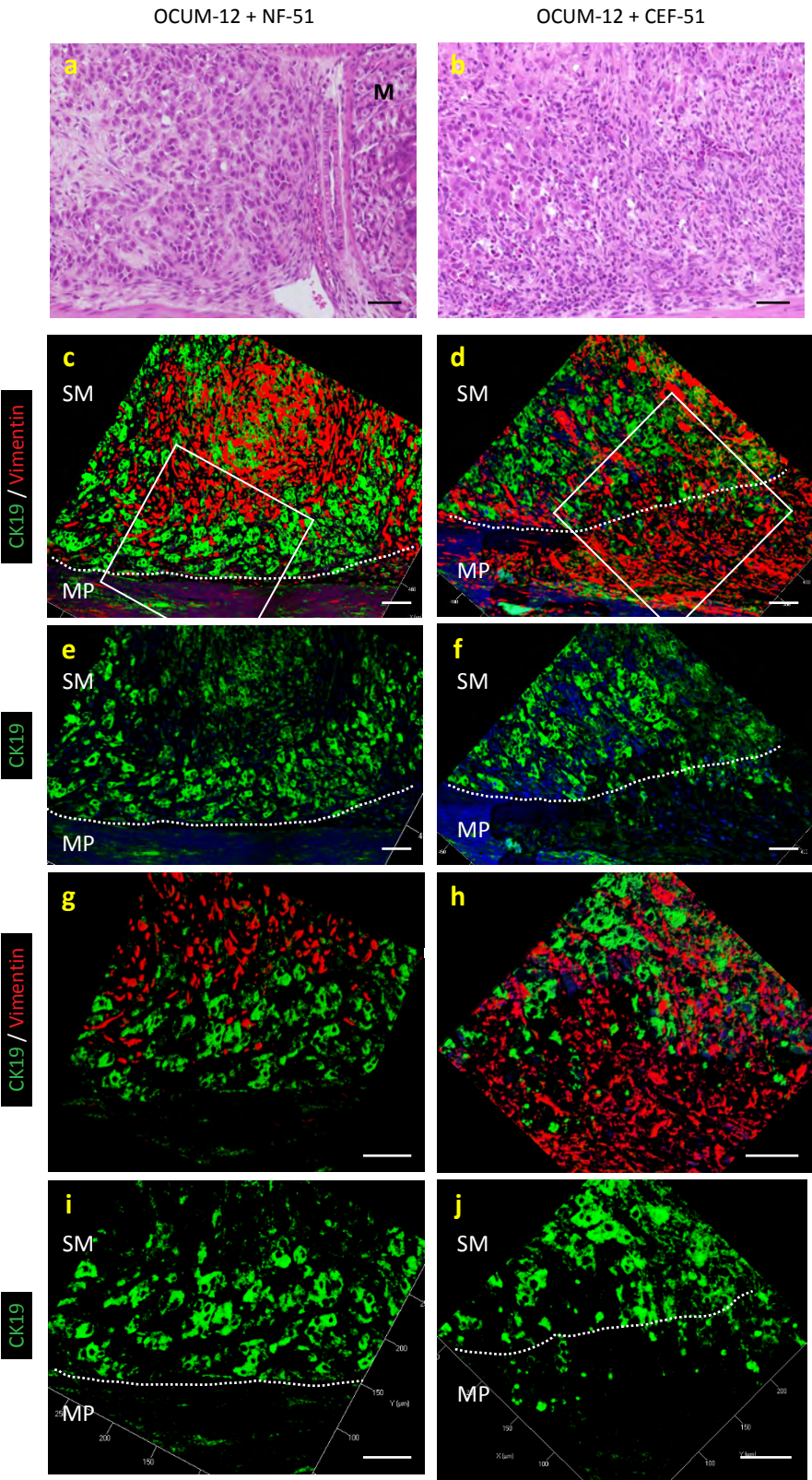

Supplement: Supplementary file 5 — Fig. S5. Tumor dissemination in the murine stomach. [file MOL2-16-166-s005.pdf]

Supplementary Figure. 6

A Mouse pancreas injection

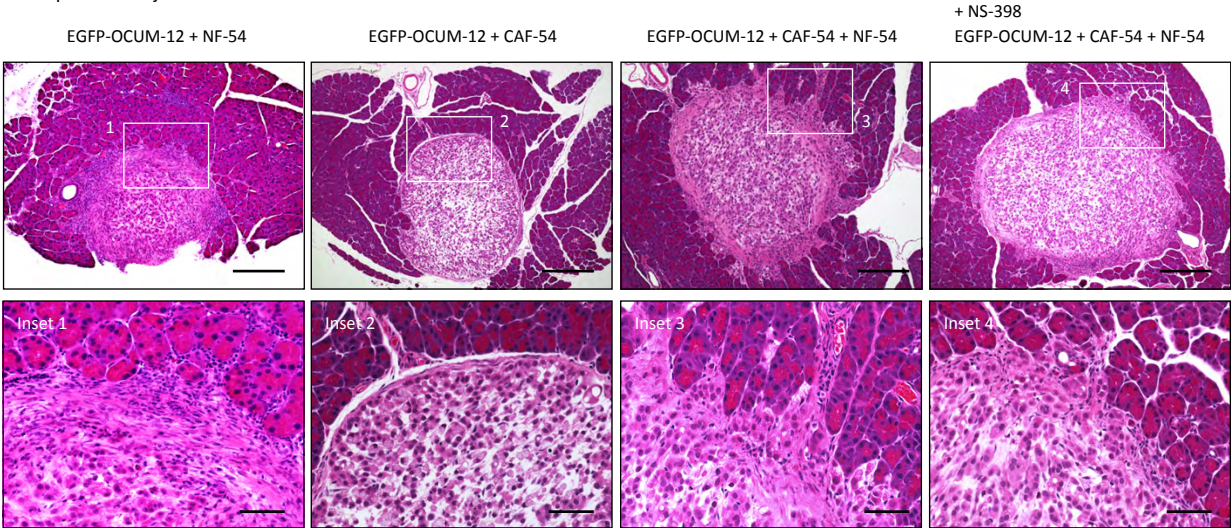

B Mouse pancreas injection

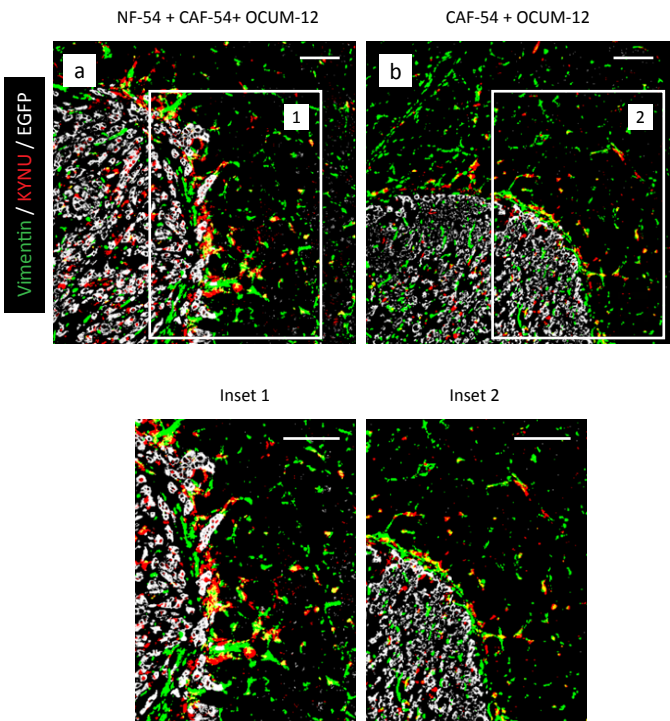

Supplement: Supplementary file 6 — Fig. S6. CEFs promote cancer cell invasion in vivo. [file MOL2-16-166-s008.pdf]

Supplementary Figure. 7

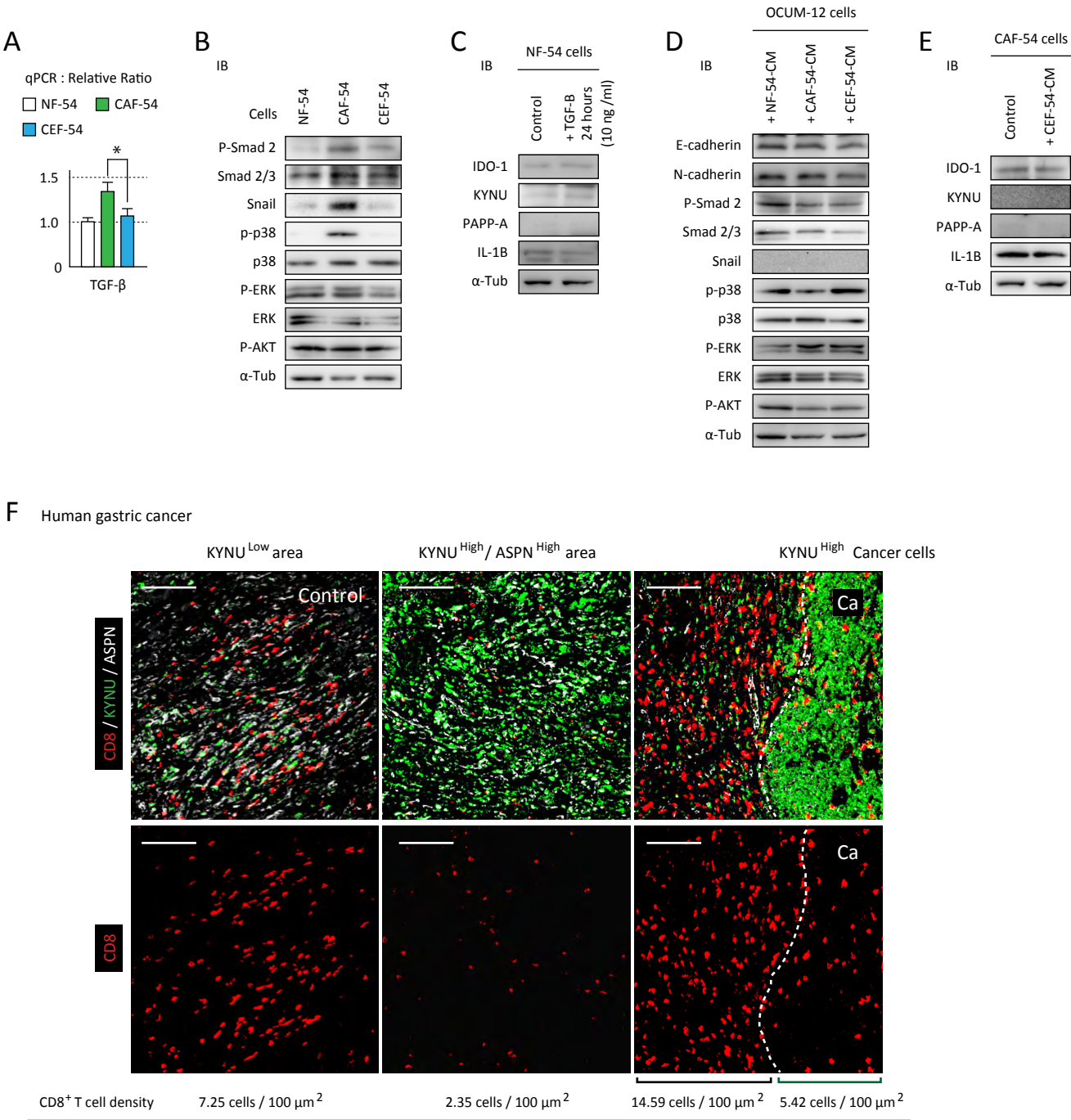

Supplement: Supplementary file 7 — Fig. S7. Evaluation of TGF‐β signaling in CEFs, and infiltration of CD8+ T cells in gastric cancer. [file MOL2-16-166-s006.pdf]

Supplementary Figure. 8

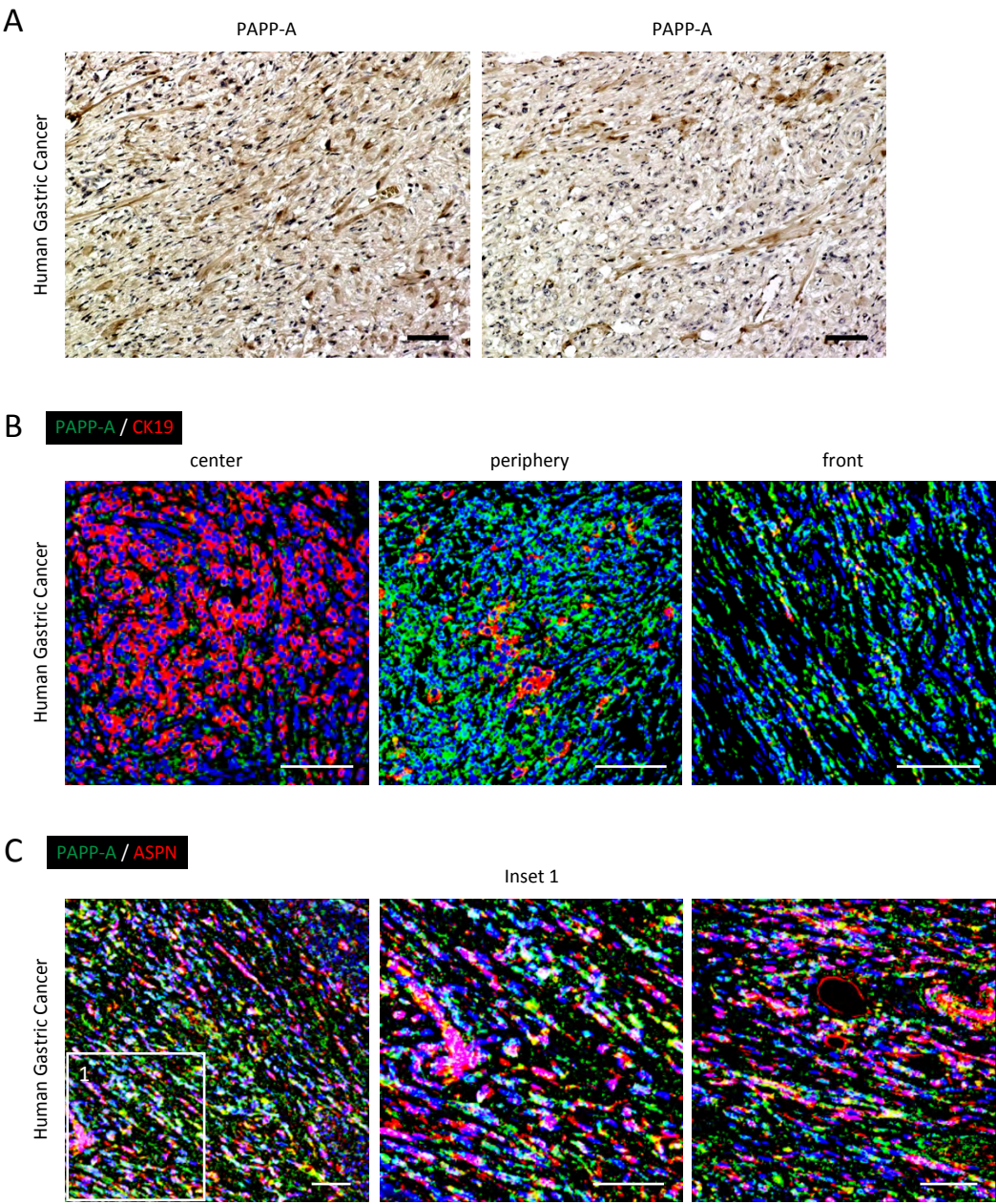

Supplement: Supplementary file 8 — Fig. S8. Immunohistochemical analysis of PAPP‐A in gastric cancer. [file MOL2-16-166-s007.pdf]
