# Supplementary material for: Cancer‐associated fibroblasts educate normal fibroblasts to facilitate cancer cell spreading and T‐cell suppression
Source: Mol Oncol. 2021 Nov 5;16(1):166–87. doi: 10.1002/1878-0261.13077 (PMC8732346; doi:10.1002/1878-0261.13077)

Supplementary Figure. 3

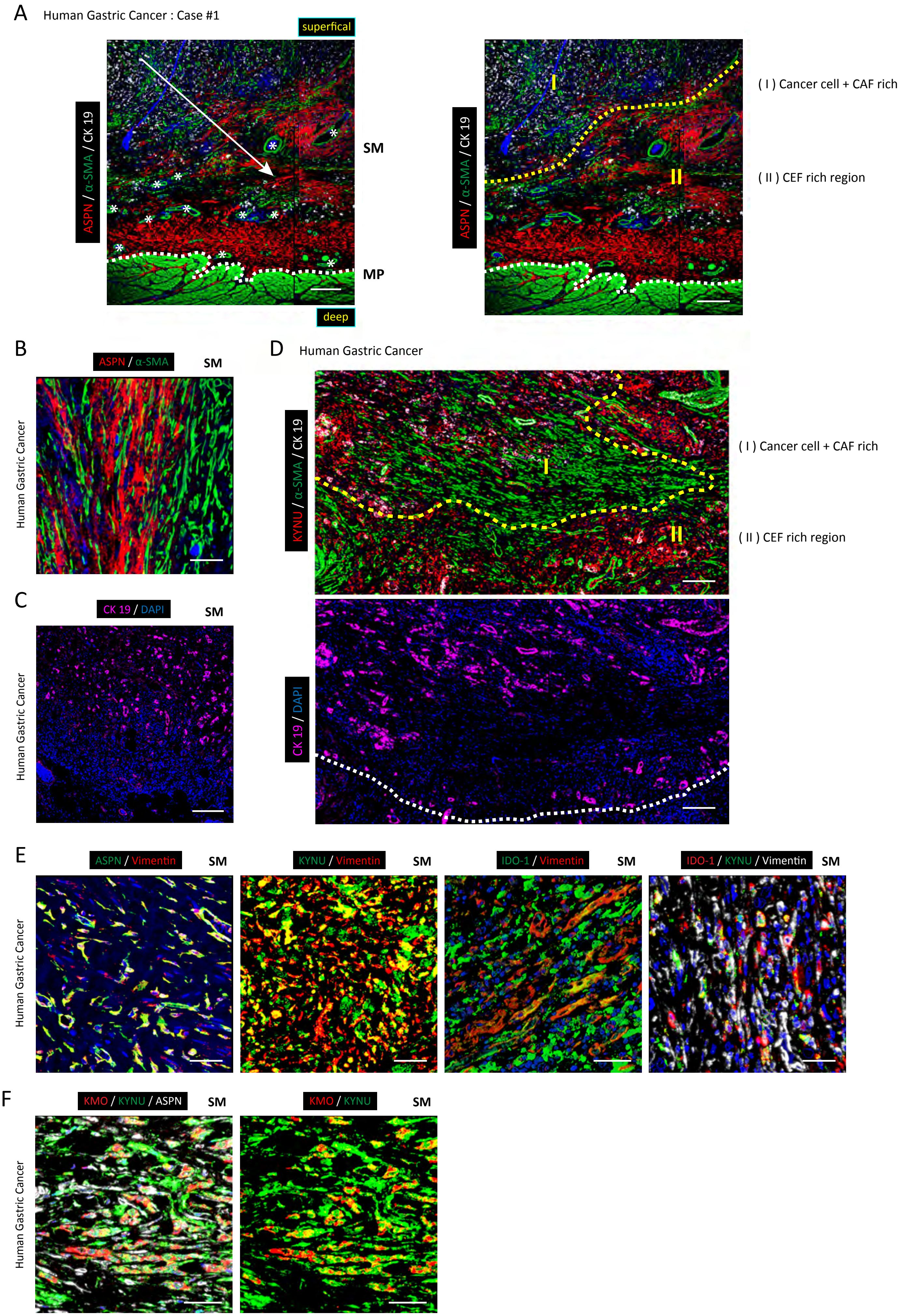

Supplement: Supplementary file 3 — Fig. S3. Localization of CEFs in gastric cancer. [file MOL2-16-166-s002.pdf]
