# Supplementary material for: Cancer‐associated fibroblasts educate normal fibroblasts to facilitate cancer cell spreading and T‐cell suppression
Source: Mol Oncol. 2021 Nov 5;16(1):166–87. doi: 10.1002/1878-0261.13077 (PMC8732346; doi:10.1002/1878-0261.13077)
Supplement: Supplementary file 9 — Table S1. Primer sequences used for quantitative PCR. [file MOL2-16-166-s010.pdf]

Table 1. Primer sequences used for quantitative PCR

Gene	Forward primers (5'-3')	Reverse primers (5'-3')
ASPN	ACCACCAACTTTATTGGAGCTT	TCACACGTGGTATGTTAGCAAGA
CRLF1	ACGGCCATAACAGCTCTGAC	TTTTGGAGGGGCCCTAGGTA
CST-1	AGGAGACCATGGCCCAGTAT	CTGGCTCTTAGTACCCGCAG
CXCL-1	AGATCATTGTGAAGGCAGGGG	CCCCTTTGTTCTAAGCCAGAAA
CXCL-6	CCCGGAAGCCCCTTTTCTAA	GTAGGCTTTCCCCCACACTC-
CXCL-8	TCCAAACCTTTCCACCCCAAA	TGCTTGAAGTTTCACTGGCAT
EGR2	CAGACAGGAGAGAGTCAGTGG	CCTTGGCGGTCATCATTTGC
FGF2	GGGTGGAGATGTAGAAGATGTGA	GCTCTTCTGTCCGCCG
GAPDH	TGACATCAAGAAGGTGGTGAAGC	CACCCTGTTGCTGTAGCCAAAT
HGF	TTTGCCTTCGAGCTATCGGG	TGATCCCAGCGCTGACAAAT
IL-1β	CAGAAGTACCTGAGCTCGCC	CATGGCCACAACAACTGACG
IL-6	GCCTTCGGTCCAGTTGCCTT	AGTGCCTCTTTGCTGCTTTCAC
KMO	CATTGGTGGTGGCTTGGTTG	TTCCAGGCCAACAGCTTTCA
KYNU	GGGATCCTAGCTGTTTTAGAGAA	CAACTGGAGGCAGATCCTGT
LIF	CTCGCCCATCACCTCATCTC	GCAGAGCTGTTTCACGCAA
PAPP-A	GGAGGGAATTCAGCGGATCA	TCCACTCTCCCCACCTTTGA
PDPN	CGCGCAAGAACAAAGTCCAA	GGCGTAACCCTTCAGCTCTT
αSMA	AGCGTGGCTATTCCTTCGTT	TGAAGGATGGCTGGAACAGG
TGF-β	CTGTCCAACATGATCGTGCG	GACACAGAGATCCGCAGTCC
TNFIP6	AGATGACCCAGGTTGCTTGG	TGGAAACCTCCAGCTGTCAC
