# Supplementary material for: Cancer‐associated fibroblasts educate normal fibroblasts to facilitate cancer cell spreading and T‐cell suppression
Source: Mol Oncol. 2021 Nov 5;16(1):166–87. doi: 10.1002/1878-0261.13077 (PMC8732346; doi:10.1002/1878-0261.13077)
Supplement: Supplementary file 10 — Supplementary Material [file MOL2-16-166-s003.docx]

**Supplementary figure**

**Fig S1. CEFs promoted cancer cell dispersion. (A)** OCUM-12 cells after incubation with NF-54-CM or CEF-54-CM as in Fig. 3B. Scale bar = 200 μm. **(B)** Biotin+ NFs were collected from mouse xenografts as in Fig 2D (F1: biotin-NF monoinjection, F2: biotin-NF + CAF co-injection), and further cultured for 24h to collect CM. Representative images of OCUM-12 cells after 4 h incubation with each CM are shown. Scale bar = 50 μm. **(C)** The same experiments as in Fig. 3E are shown by graphic overlay of trajectory and captured images. Scale bar = 200 μm. **(D)** Boyden chamber assay of OCUM-12 cells. Representative fields from each experiment are shown. Results represent three independent experiments, and are expressed as the relative ratio to control cells. **P* <0.05. **(E)** Velocity of the migration of single OCUM-12 cells. Left: Cells were incubated with fibroblast CM at a low cell density (1 × 10^4^ / 35 mm dish), and time-lapse images were captured at 10-min intervals for 600 min. Right: Comparison of the migration velocity of individual cells. **P* <0.05. **(F)** CEF-CM was added to OCUM-12 cells with or without Y27632 (100 μM, Rho kinase inhibitor) or GM6001 (20 μM, broad-spectrum MMP inhibitor). Neither inhibitor suppressed OCUM-12 cell dispersion. Scale bar = 200 μm. **(G)** OCUM-12 cells were incubated with CM from NF-51, CAF-51, or CEF-51 for 24 h. Cells were fixed and subjected to immunofluorescence staining with an anti-Ki67 antibody (green) and DAPI (blue). Merged images of fluorescence are shown. Right: Comparison of Ki67 labeling (%). Scale bar = 50 μm. Statistical significance was calculated by a one-way ANOVA followed by Tukey’s post-hoc tests.

**Fig S2. Activation of the KYNU pathway by ASPN, and the effects of cancer cell CM on NFs. (A)** CD8^+^ T cells were incubated with NF-54-CM or CEF-54-CM for 2 days and subjected to FACS analysis. Dot plots of PI and CD8 staining (left), and histograms of PI staining (right). Arrows indicate dead cells. In the dot plots, the numbers indicate the percentage of cells in each fraction. **(B)** The purity of isolated biotin-labeled NFs in Fig. 4G was tested by immunofluorescence. Biotin+ NFs were collected from a mixture of CAFs with streptavidin coated microbeads and were passaged through separating columns. Separated biotin+ NFs or flow-through cells were fixed and stained with streptavidin (red) and DAPI (blue). The purity of biotin+ NFs was almost 100% (200 cells were counted). Scale bar = 50 μm. **(C)** ASPN was knocked down in HSC-43 ASPN cells by siRNA (two siRNAs were used as described in the Materials and Methods. The control indicates nonspecific siRNA -treated cells. Cell lysates were subjected to immunoblotting. **(D)** Immunohistochemical analysis of CXCL-6 in human gastric cancer specimens. Intense staining of CXCL-6 was observed in cancer cells (diffuse-type gastric cancer specimen). **(E)** NF-54 cells were treated with CM from two gastric cancer cell lines separately for 48h, and cell lysates were subjected to western blot analysis as indicated.

**Fig S3. Localization of CEFs in gastric cancer. (A)** The left panel shows a low magnification of the area in Fig 5A (case #1). Asterisks indicate blood vessels. Right: The dotted line indicates the border of cancer cells with CAF rich region (I) and the CEF-rich region (II). Scale bar = 500 μm. SM: submucosal space. MP: muscle layer. **(B)** Immunostaining of a human gastric cancer specimen with anti-ASPN and α-SMA. Scale bars = 50 μm. **(C)** Anti-cytokeratin 19 (magenta) and DAPI (blue) of the area in Fig 5B, left panel. Scale bar = 200 μm. SM: submucosal space. **(D)** Upper: A human gastric cancer specimen was immunostained with anti-KYNU (red), anti-αSMA (green) and anti-cytokeratin19 (white). The yellow dotted line indicates the border of cancer cells with CAF rich region (I) and the CEF rich region (II). The bottom panel shows staining of the same area as in the upper panel anti-cytokeratin 19 (magenta) and DAPI (blue). The white dotted line indicates the invasive front of cancer cells. Scale bar = 200 μm. **(E, F)** Gastric cancer specimens showing co-localization of vimentin with ASPN, KYNU or IDO-1 **(E)**, and KMO with KYNU and ASPN **(F)**. Scale bar = 50 μm.

**Fig S4. Evaluation of cancer cells dispersion by Delaunay triangulation plots. (A)** Cell dispersion was analyzed by Delaunay triangulation plots. Upper panels: The irregular expansion of Delaunay triangles as estimated from the averaged area of the regular triangles (white). The larger triangles indicate that cell-cell interactions are weaker. Bottom: Original photos used for the analysis. The cyan dots indicate the centroids of cell body. The upper panels show the plot results within the white frame. Scale bar = 100 μm. **(B)** Representative image of OCUM-12 cells incubated with L-CEF-7d-50-CM. Scale bar = 100 μm. Right; Expressions of CEF marker genes in L-CEF-7d-50 was examined by western blotting.

**Fig S5. Tumor dissemination in the murine stomach.** OCUM-12 cells (2 x 10^5^) were transplanted in mouse gastric wall together with either NF-51 or CEF-51 (2 x 10^5^). Mice were sacrificed at day 8, and xenografts were formalin-fixed and paraffin-embedded (FFPE) and subjected for immunostaining with anti-CK19 (green) and anti-Vimentin (red) antibody to detect cancer cells and fibroblasts, respectively (c-j). Maximum cut surface was shown in each xenograft. The upper panels are H&E staining (a, b). M: mucosal layer. The boxed areas (c, d) are enlarged in the bottom panels (i, j). Five mice were examined in each group, and representative images are shown. Dotted line indicates border between SM: submucosa and MP: muscle layer. Scale bars = 50 μm.

**Fig S6. CEFs promote cancer cell invasion *in vivo*. (A)** H&E staining of mouse pancreas xenografts described in Fig 6E. The boxed areas in the upper panels are enlarged in the bottom panels. Scale bar = 200 μm (upper panels) and 50 μm (bottom panels). **(B)** Evaluation of KYNU^+^ fibroblasts in xenografts. Tumors of EGFP-OCUM-12 cells co-injected with a mixture of NFs and CAFs (a), or with CAFs alone (b) were immunostained with anti-KYNU (red), anti-vimentin (green) and anti-EGFP (white). The boxed area is enlarged in the bottom panels. Scale bar = 50 μm. Expression of KYNU in fibroblasts was rarely detected (b).

**Fig S7. Evaluation of TGF-β signaling in CEFs, and infiltration of CD8^+^ T cells in gastric cancer. (A)** Comparison of the expression of TGF-β in NFs, CAFs and CEFs by qRT-PCR. Statistical significance was calculated by a one-way ANOVA followed by Tukey’s post hoc tests (n=3, *P < 0.05). **(B)** Activation of TGF-β signaling in NFs, CAFs and CEFs was examined by western blotting with the indicated antibodies. **(C)** NF-54 cells were incubated with recombinant TGF-β (10 ng/mL) for 24h. Cell lysates were prepared and subjected to western blotting. **(D)** Activation of EMT-related genes in OCUM-12 cells. OCUM-12 cells were incubated with CM from NF-54, CAF-54, or CEF-54 for 24h, and were subjected for western blot analysis. **(E)** CAF-54 cells were cultured with CEF-54-CM for 24h, and gene expression was examined by western blotting. **(F)** Human gastric cancer specimens were immunostained with anti-CD8 (red), anti-KYNU (green) and anti-ASPN (white). Representative images are shown. Right: As a control case, the boundary of the stroma and KYNU^high^ cancer cells nest is shown. White dotted line indicates boundary of cancer cell nest (Ca). The values indicate the density of CD8 positive T cells. Scale bars = 100 μm.

**Fig S8. Immunohistochemical analysis of PAPP-A in gastric cancer. (A)** Human gastric cancer specimens were immunostained with an anti-PAPP-A antibody. Positive staining for PAPP-A was mainly observed in stromal fibroblastic cells. **(B)** The same specimen as in A was subjected to co-immunostaing with anti-PAPP-A (green) and anti-cytokeratin19 (red). Representative images of the center, periphery or invasive front of the tumor were shown. Scale bar = 100 μm. DAPI staining is shown in blue. **(C)** The same specimen was immunostained for PAPP-A (green) and ASPN (red). The boxed area is enlarged in the middle panel. Scale bar = 50 μm.
